# Modulating Aluminum Solvation with Ionic Liquids for Improved Aqueous-Based Aluminum-Ion Batteries

**DOI:** 10.1021/acsaem.3c01745

**Published:** 2023-11-22

**Authors:** Abhishek Lahiri, Shaoliang Guan, Arunabhiram Chutia

**Affiliations:** † Department of Chemical Engineering, Brunel University London, Uxbridge UB8 3PH, U.K.; ‡ School of Chemistry, Cardiff University, Cardiff CF10 3AT, U.K.; § HarwellXPS, Research Complex at Harwell, Rutherford Appleton Laboratory, Didcot OX11 0FA, U.K.; ∥ School of Chemistry, University of Lincoln, Brayford Pool, Lincoln LN6 7UY, U.K.

**Keywords:** Al-ion batteries, solvation structure, ionic liquids, aqueous electrolytes, density functional theory, spectroscopy

## Abstract

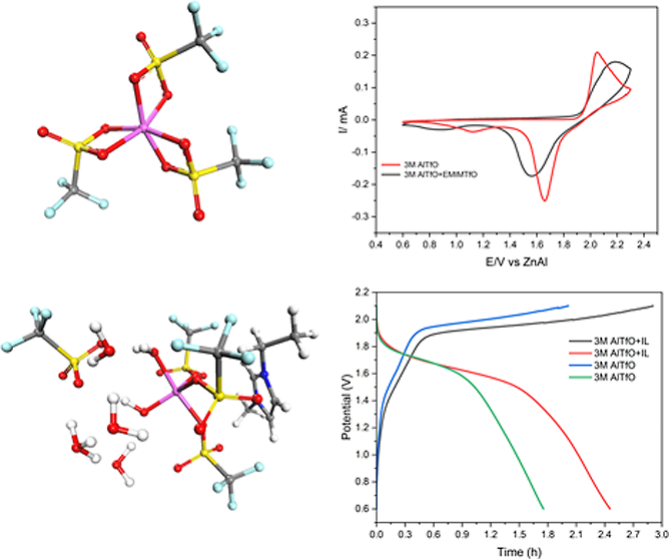

Aqueous-based Al-ion batteries are attractive alternatives to Li-ion batteries due to their safety, high volumetric energy density, abundance, and recyclability. Although aluminum-ion batteries are attractive, there are major challenges to overcome, which include understanding the nature of the passive layer of aluminum oxide on the aluminum anode, the narrow electrochemical window of aqueous electrolytes, and lack of suitable cathodes. Here, we report using experiments in conjunction with DFT simulations to clarify the role of ionic liquids (ILs) in altering the Al solvation dynamics, which in turn affects the aluminum electrochemistry and aqueous-based battery performance significantly. DFT calculations showed that the addition of 1-ethyl-3-methylimidazolium trifluoromethylsulfonate (EMIMTfO) changes the aluminum solvation structure in the aqueous (Al(TfO)_3_) electrolyte to lower coordinated solvation shells, thereby influencing and improving Al deposition/stripping on the Zn/Al alloy anode. Furthermore, the addition of an IL reduces the strain in manganese oxide during intercalation/deintercalation, thereby improving the Zn/Al-MnO_*x*_ battery performance. By optimizing the electrolyte composition, a battery potential of >1.7 V was achieved for the Zn/Al-MnO_*x*_ system.

## Introduction

There is a great demand for developing low-cost electrochemical energy storage devices. Although lithium-ion batteries (LIBs) have dominated the market, the cost, safety, availability of lithium, and recycling of the batteries are some of the main issues, which has led to new research for alternate battery chemistries/technologies.^1−4^ Among the low-cost alternatives, sodium, magnesium, zinc, and aluminum batteries have particularly attracted interest in recent years. However, compared to all the low-cost alternatives, aluminum-based batteries stand out as aluminum is readily available and have the highest theoretical volumetric capacity of 8056 mA h cm^–3^ and a modest gravimetric capacity of 2981 mA h g^–1^.^5^ Furthermore, the technology for recycling aluminum is well-known^6^ which makes it a prospective battery material, conferring well with the principles of circular economy.

Aluminum batteries have been researched for the last 50 years with aluminum as an anode and different metal/metal oxides, sulfides, and carbides as cathodes.^7^ Between 1980 and 2005, aluminum batteries were mainly researched in molten salt electrolytes where dendrite formation was a major issue along with the dissolution of cathodes.^7^ However, recent results on aluminum batteries with molten salt have shown promising perspective.^8^ However, for molten salt electrolytes, a temperature >150 °C is required which limits its application; therefore, both aqueous and nonaqueous-based Al batteries have been researched in the last couple of years.

For the effective Al deposition/stripping and intercalation and deintercalation processes, the aluminum speciation in the electrolyte is an important factor to control. In ionic liquid (IL)-based electrolyte (for example, AlCl_3_/EMIMCl), AlCl_4_^–^ was shown to be responsible for intercalation/deintercalation in the carbon cathode and Al_2_Cl_7_^–^ for aluminum deposition and stripping on the Al anode, respectively, which led to a very fast charging capability.^9^ In comparison, Al deposition/stripping has recently been achieved in aqueous Al batteries using various electrolytes.^10−13^ Among them, aluminum triflate (Al(TfO)_3_) and aluminum *bis*(trifluoromethanesulfonyl)amide electrolytes with a Zn/Al anode and MnO_2_ cathode show a storage capacity of >400 mA h g^–1^.^10−12^ In the Zn/Al system, the authors argued that alloying of Al with Zn inhibits the alumina passivation layer and therefore can compete with the hydrogen evolution reaction, which leads to a better Al deposition/stripping as well as show a better battery performance.^11^ However, recently, using spectroscopic, electrochemical, and theoretical studies, it was shown that in the Al(TfO)_3_ system that the hydrogen evolution reaction hinders the aluminum deposition/stripping and results in the formation of hydrogen bubbles.^14^ For the MnO_2_ cathode case, it has been shown that the dissolution of the cathode takes place in the electrolyte.^15^

Here, we show that the addition of 1-ethyl-3-methylimidazolium trifluoromethylsulfonate (EMIMTfO) to Al(TfO)_3_ improves the Al deposition/stripping process on Zn. EMIMTfO is fully miscible in water and has been shown to change the electrode/electrolyte interface, significantly depending on the concentration of water, which also affects the electrochemical window.^16^

## Experimental Section

The ILs, [EMIM]TfO (99%), was purchased from IoLiTec, Germany. Aluminum triflate (thermoscientific) (99%), manganese sulfate (99%), sodium sulfate (99%), and aniline (>98%) were purchased from Fisher Scientific.

The MnO_2_ was electrochemically synthesized in a two-electrode setup with graphite paper as an anode and carbon sheet as a cathode. A constant current density of 4 mA cm^–2^ was passed between the electrodes from a solution of 0.2 M Na_2_SO_4_ and 5 mM MnSO_4_ for different times between 10 and 30 min. After the deposition, the samples were washed with deionized water several times to remove any impurities. The mass loading ranged between 1 and 2.3 mg cm^–2^.

Electrochemical measurements were carried out in a split cell consisting of the MnO_2_/MnO_2_-polyaniline electrode as the working electrode, Zn foil as counter and reference electrodes, and 3 mol kg^–1^ Al(TfO)_3_ or 3 M Al(TfO)_3_ with different concentrations of EMIMTfO. CV was performed in the potential range 0.5–2.3 V versus Zn/Al at 1 mV s^–1^ scan rate by using a Biologic VMP 3e potentiostat/galvanostat. The galvanostatic charge/discharge cycling tests were carried out in a split cell with the Zn anode, a Whatman separator, and the MnO_*x*_ cathode with different aluminum electrolytes by using a battery tester nanocycler from nanobase at 100 mA g^–1^ current density.

The morphology of the MnO_2_ samples was investigated by scanning electron microscopy (SEM, JEOL JSM6610LV). IR were recorded on Shimadzu IR spirit.

XPS analysis was performed using a Kratos Axis SUPRA XPS fitted with a monochromated Al kα X-ray source (1486.7 eV), a spherical sector analyzer, three multichannel resistive plate, and 128 channel delay line detectors. All data were recorded at 150 W and a spot size of 700 μm × 300 μm. Survey scans were recorded at a pass energy of 160 eV, and high-resolution scans were recorded at a pass energy of 20 eV. Electronic charge neutralization was achieved by using a magnetic immersion lens. Filament current = 0.27 A, charge balance = 3.3 V, and filament bias = 3.8 V. All sample data were recorded at a pressure below 10^–8^ Torr and at room temperature of 294 K. Data were analyzed using CasaXPS v2.3.20PR1.0, and the spectra were calibrated with C 1s peak at 284.8 eV.

## Computational Details

The density functional theory (DFT)-based quantum chemical calculations were performed by using the ORCA 5.0.3 code.^100−102^ For all these calculations, the Becke three-parameter hybrid exchange and correlation functional (B3LYP) in combination with Grimme’s D3 correction were used. The geometries of aluminum triflate [Al·(OTf)_3_], [Al·(OTf)_3_]·6H_2_O, and five different models of [Al·(OTf)_3_]·6H_2_O with imidazolium triflate molecules at different positions were optimized using the default Broyden–Fletcher–Goldfarb–Shano algorithm. The harmonic vibrational frequency calculations were performed on the optimized geometries to ensure that all the geometries have real frequencies. The convergence criteria for the total energy change, maximum gradient, root-mean-square (RMS) gradient, maximum displacement, and RMS displacement were set to 5 × 10^–6^ Ha, 3 × 10^–4^ Ha/Bohr, 1 × 10^–4^ Ha/Bohr, 4 × 10^–3^ Bohr, and 2 × 10^–3^ Bohr, respectively. In all of the calculations, the def2-TZVP basis set was used.

## Results and Discussion

**Figure 1:**
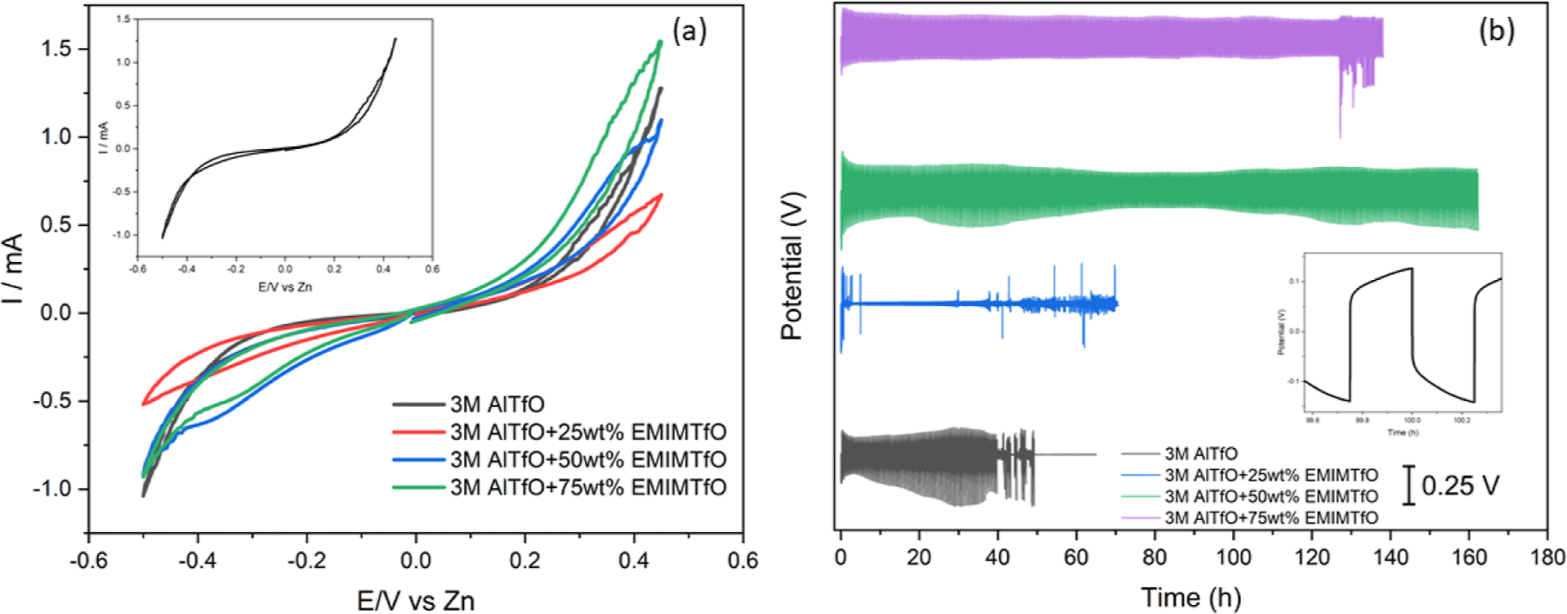
(a) CV of AlTfO with and without EMIMTfO on Zn. (b) Deposition/stripping of Al in a Zn/Zn symmetric cell from different electrolytes.

Figure 1a compares the cyclic voltammetry (CV) of the Al(TfO)_3_ electrolyte with the addition of EMIMTfO on Zn. It is evident that the addition of IL > 25 wt % leads to a clear Al deposition peak at −0.3 V and an Al stripping wave at 0.3 V on Zn. The inset shows the first cycle without the IL from which a nucleation loop is observed at −0.4 V. This can be attributed to Al deposition. On continuation of CV cycling (Figure S1), prominent Al deposition/stripping peaks at −0.32 and +0.3 V can be seen in the Al(TfO)_3_ electrolyte, whereas in the presence of IL, the Al deposition/stripping peaks shift to −0.37 and +0.3 V, respectively. This indicates that the passive oxide layer might have dissolved electrochemically/chemically in the electrolyte.

**Figure 2:**
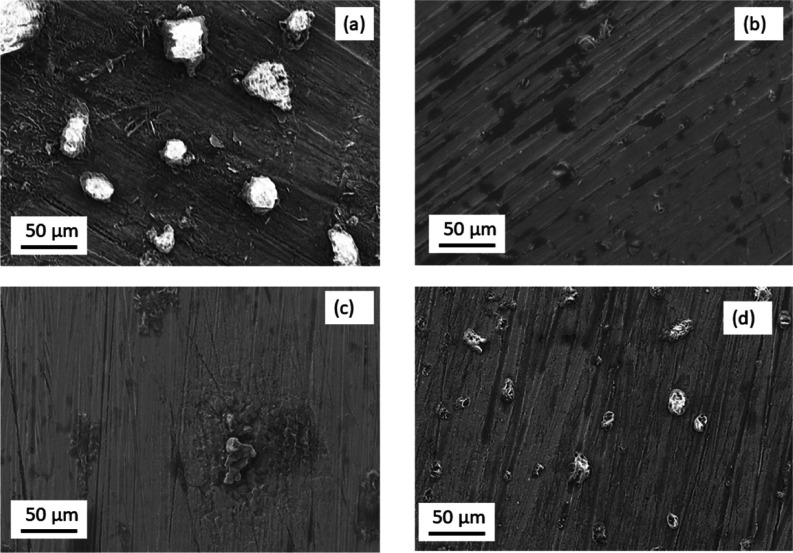
(a) SEM of Al deposit on Zn after cycling in the Zn/Zn symmetric cell in 3 M AlTfO. (b) SEM of Al deposit on Zn after cycling in the Zn/Zn symmetric cell in 3 M AlTfO + 25 wt % EMIMTfO. (c) SEM of Al deposit on Zn after cycling in the Zn/Zn symmetric cell in 3 M AlTfO + 50 wt % EMIMTfO. (d) SEM of Al deposit on Zn after cycling in the Zn/Zn symmetric cell in 3 M AlTfO + 75 wt % EMIMTfO.

To further understand the implication of Al deposition/stripping peaks on Zn, galvanostatic experiments were performed for a long time. Figure 1b compares the Al deposition/stripping from different electrolytes in a Zn/Zn symmetric cell from which it is evident that the presence of EMIMTfO at 50 wt % and above leads to stable deposition/stripping of Al on Zn. The inset in Figure 1b shows one of the deposition/stripping cycles in 3 M Al(TfO)_3_ + 50 wt % EMIMTfO. However, at higher current densities, even with the addition of IL, instability in the deposition/stripping was observed. The SEM of Al deposition/stripping without IL (Figure 2a) and at a lower concentration of IL (Figure 2b) resulted in the formation of sulfurous particles (Figures S2, S3), which might be due to decomposition of the triflate ion.^17,18^

With 50 wt % EMIMTfO added to the electrolyte, no such particles were observed in the SEM (Figures 2c and S4). Interestingly, although stable Al deposition/stripping was observed with 3 M Al(TfO)_3_ + 75 wt % EMIMTfO electrolyte for 120 h, sulfurous particles were still observed on Zn (Figures 2d and S5). Therefore, it appears that the sudden spikes in the potential observed in Figure 1b might be related to the decomposition of triflate anions, leading to the deposition of sulfurous compounds.

**Figure 3:**
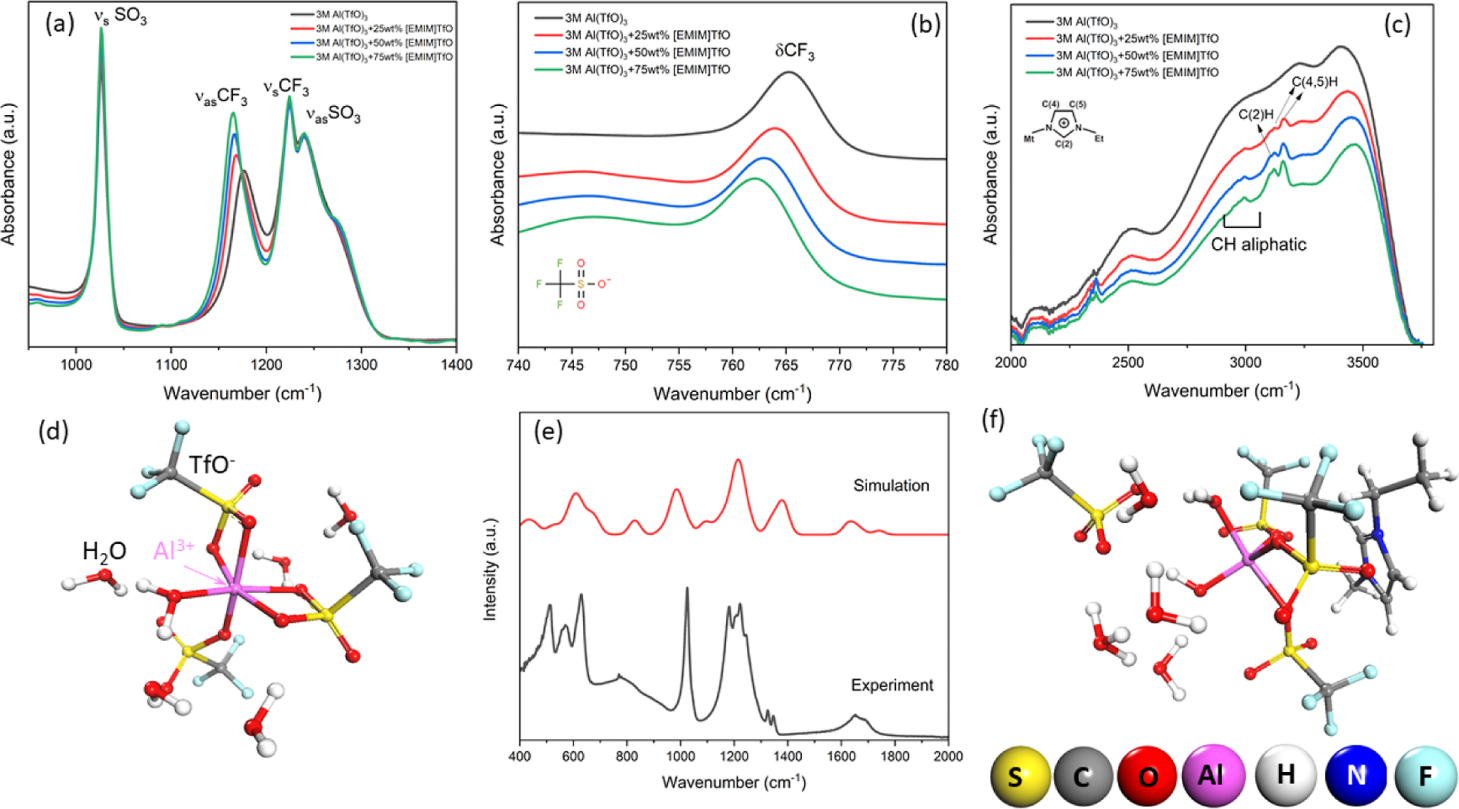
FTIR spectra of AlTfO with different concentrations of EMIMTfO (a) between 900 and 1400 cm^–1^, (b) between 740 and 780 cm^–1^, and (c) between 2000 and 4000 cm^–1^. (d) Geometries of Al(TfO)_3_·6H_2_O with all the TfO^–^ ions bicoordinated to the Al^3+^ ion in the initial structures. (e) Comparison between experimental and simulated IR spectra for AlTfO in water. (f) Fully relaxed geometries of Al(TfO)_3_·6H_2_O with EMIMTfO.

The change in CV and the stability in Al deposition/stripping in the presence of IL indicate that Al solvation in the electrolyte influences the process. Fourier-transformed infrared (FTIR) spectroscopy was then used to further investigate the factors influencing Al solvation in the electrolyte. The FTIR in Figure 3a–c shows the various regions of the spectra. From Figure 3a,b, it is evident that the addition of an IL leads to a shift in the CF_3_ vibration to lower wavenumbers, which can be associated with the change in Al coordination. In the wavenumber region between 2000 and 3600 cm^–1^, the addition of IL leads to peaks related to the imidazolium cation.^15^ To better understand the coordination of Al in the aqueous electrolyte and the effect of EMIM^+^TfO^–^ ions, DFT-based quantum chemical calculations were performed. Previous theoretical reports suggested that the TfO^–^ ions can have bidentate or monodentate coordination with the Al^3+^ ion.^19^ Therefore, in the first step, we explored the geometries of mono- and bidentate Al(TfO)_3_. As shown in Figure S6, in the case of the monodentate Al(TfO)_3_ molecule, two of the TfO^–^ ions changed from monodentate to bidentate coordination while the third TfO^–^ ion remained monocoordinated (Figure S6a). However, in the bidentate Al(TfO)_3_ molecule after optimization, the TfO^–^ ions remain coordinated with the Al^3+^ ions via two oxygen ions (Figure S6b).

A probable reason for one of the TfO^–^ ions to remain monodentate leading to the formation of this hybrid model could be related to steric hindrance posed by the other two TfO^–^ ions. To understand the stabilities of these two models, we calculated the interaction energy (IE) per species (*E*_IE/species_), using eq 1
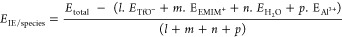
1Here, *E*_TfO^–^_(*l*), *E*_EMIM^+^_(*m*), *E*_Al^3+^_(*p*), and *E*_H_2_O_(*n*) are the energies of the triflate, 1-ethyl-3-methyl imidazolium, Al^3+^ ions, and water molecule, respectively. The *l*, *m*, *n*, and *p* within the parentheses refer to the number of these species in the molecular system under consideration. Finally, the *E*_total_ is the energy of the total system. We note that the lower the *E*_IE/species_, the more stable the system. The calculated (*E*_IE/species_) showed that the relative *E*_IE/species_ between these two models is ∼0.06 eV, which is comparable. Therefore, we performed the rest of this study using the hybrid Al(TfO)_3_ model. We note that for these two models *n* = *m* = 0.

Another study recently reported the presence of six water molecules in proximity of the Al(TfO)_3_ molecule;^14^ therefore, in the next step, we included six water molecules around the hybrid Al(TfO)_3_ model. As shown in Figure 3d, in the fully relaxed Al(TfO)_3_·6H_2_O geometry, we found that a network of hydrogen bonds between the water molecules and the TfO^–^ ions was formed. Further to this, one of the water molecules coordinated to the Al^3+^ ion with an Al–O interatomic distance of 1.83 Å, which is comparable to the Al–O interatomic distance [(Al–O)_water_] for mono (1.84 Å) and bidentate (1.96 Å) TfO^–^ ions. The Mayer bond orders for (Al–O)_water_ and (Al–O)_monodentate_ are 0.77 and 0.71, respectively, which confirms that this water molecule is coordinated to the Al^3+^ center via a chemical bond. The theoretical IR spectrum for Al(TfO)_3_·6H_2_O is shown in Figure 3e, and the major IR peaks are summarized in Table 1, which shows a close agreement with the experimental IR spectrum, and Figure 3d shows the relaxed structure. We note that due to the addition of six water molecules to the Al(TfO)_3_ model, there were no changes in the manner in which the TfO^–^ ions interact with the Al^3+^ ion.

**Table 1:** Comparison of Experimental and DFT Simulation of IR Vibrational Modes

wavenumber (cm^–1^)		
exp	DFT	vibrational modes
Al(TfO)_3_·6H_2_O		
1694	1740.2	HOH scissoring in water
1652	1633.96	HOH scissoring in water
1346/1326	1378.53	S–O stretching
1181/1221	1211.24	CF_3_ asymmetric stretching
1023	988.68	O–S symmetric stretching
780	764.71/769.64/771.66	water wagging in combination with CF_3_–SO_3_ stretching
	828.81	
629	673.6/625.69	water wagging
M5		
1632	1763.59	HOH scissoring in water
1574	1605.59	HOH scissoring in water
1282	1281.53	combination of stretching between CF_3_–SO_3_
1241	1245.12	combination of stretching between CF_3_–SO_3_
1225	1205.14/1213.97/1222.94/1229.30/1232.66	combination of stretching between CF_3_–SO_3_
1166	1129.49/1132.94/1166.86	CF_3_ stretching in combination with CH = CH symmetric stretching in imidazole
1025.5	999.76	SO_3_ symmetric stretching in combination with CF_3_
765	752.29/756.88/758.23	water wagging in combination with CF3 symmetrical stretching with imidazolium

Next, we investigated the effect of the presence of the EMIM^+^ and TfO^–^ ions on the Al(TfO)_3_·6H_2_O geometry. We considered three cases, i.e., the EMIM^+^ and TfO^–^ ions were (i) placed close to each other on one side of the Al(TfO)_3_·6H_2_O model, (ii) placed on either side of the Al(TfO)_3_·6H_2_O model, and (iii) placed relatively further away than case (i) but closer than case (ii). These three models are referred to as M1, M2, and M3, respectively (see Figure S7a–c). In M1 and M2, with the addition of EMIM^+^ and TfO^–^ ions, the water molecule coordinated to the Al^3+^ ions dissociated to give an H_3_O^+^ species. In M3, even though no dissociation of the water molecules was seen, there was however a change in the solvation around the Al^3+^ ion, i.e., the coordination of two of the TfO^–^ ions to the Al^3+^ ion was transformed from a bidentate to a monodentate coordination. The theoretical IR spectrum showed closer agreement with experimental data for M2 as compared to M1 and M3 (Figures S8, S7). From these three models, we concluded that the presence of EMIM^+^ and TfO^–^ ions may lead to the dissociation of water molecules and/or change the solvation around the Al^3+^ ion. The calculated *E*_IE/species_ of M1, M2, and M3 are, respectively, −5.19, −5.20, and −5.15 eV, which is more positive than the Al(TfO)_3_·6H_2_O system with *E*_IE/species_ of −5.70 eV. This reveals that with the addition of the EMIM^+^ and the TfO^–^ ions, these models (M 1–3) became relatively unstable as compared to the Al(TfO)_3_·6H_2_O system, which we believe results in the change of the solvation environment around the Al^3+^ ion as evident from the calculated geometries. We saw that in the M 1–3 models, the Al^3+^ ion is bonded to the triflate ions and surrounded by a network of hydrogen-bonded water molecules, which may also influence how the EMIM^+^ and TfO^–^ ions affect the molecular environment around the Al^3+^ ion. Therefore, in the next step, we considered two more models (M4 and M5) derived from the M2 model above (see Figure S7d,e). In the M4 model, like M2, the EMIM^+^ and TfO^–^ ions were placed away from each other, however, all the six water molecules were moved closer to the TfO^–^ ion. In the M5 model, we used a similar configuration to M4 but with five water molecules close to TfO^–^ and another close to the EMIM^+^ ion. The fully relaxed geometries, as shown in Figure S7d,e, revealed that this led to the dissociation of a water molecule to give H_3_O^+^ species and to form an Al–OH bond and another water molecule moved closer to the Al^3+^ ion. Additionally, in M4 two out of three TfO^–^ ions formed monodentate coordination while in M5, all the TfO^–^ ions formed monodentate bonds. For clarity of the readers, we have included the coordinates of the fully relaxed geometries of all the models in the Supporting Information. The *E*_IE/species_ revealed comparable binding energies with the M2 model but M5 (*E*_IE/species_ = −5.28 eV) was relatively most stable (Figure 3f). A comparison of the theoretical IR spectrum for M5 shows a closer agreement with the experimental results (Table 1). From these results, we drew the following conclusions i.e., (a) water molecules may coordinate with the central Al^3+^ ion, which when deprotonated can lead to the formation of AlOH species and (b) the presence of EMIM^+^ and TfO^–^ ions may lead to the formation of H_3_O^+^ species and also change the solvation environment of the Al^3+^ ion. Therefore, the change in the Al solvation clearly affected the deposition/stripping of Al on Zn, as shown in Figure 1.

**Figure 4:**
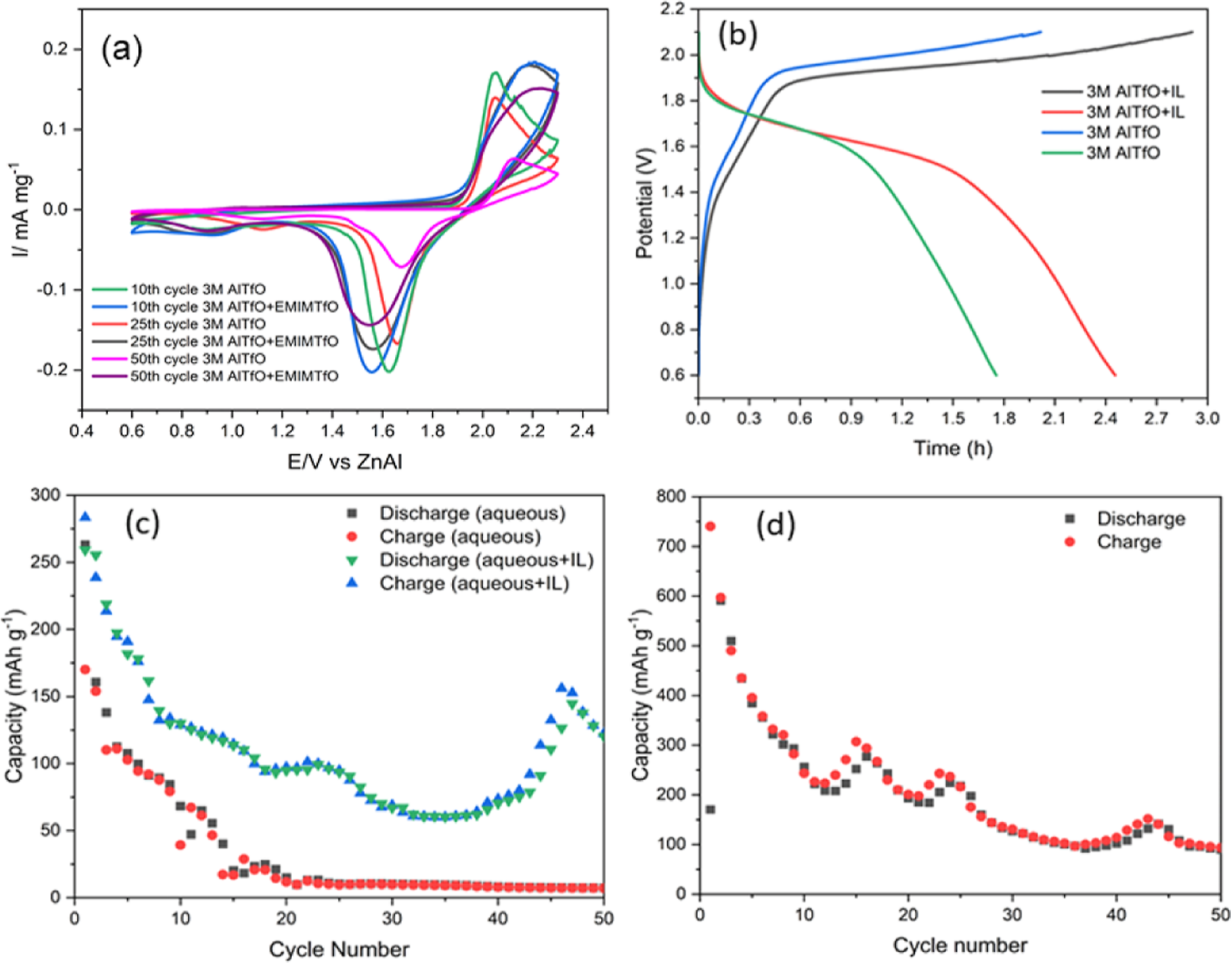
(a) CV of 3 M AlTfO and 3 M AlTfO + 50 wt %EMIMTfO on electrodeposited MnO_2_. (b) Comparison of charge–discharge in aqueous and aqueous + IL electrolytes. (c) Charge–discharge cycles in the ZnAl/MnO_2_ battery in two different electrolytes cycled at 100 mA/g. (d) Charge–discharge cycles in the ZnAl/MnO_2_ battery in 3 M AlTfO + 50 wt %EMIMTfO + 0.1 M MnTfO cycled at 100 mA/g.

Based on the above solvation studies, the influence of IL on the MnO_*x*_ cathode was also assessed. Figure 4a shows the CV of aqueous and 3 M Al(TfO)_3_ + 50 wt % EMIMTfO electrolyte on the MnO_*x*_ cathode. For aqueous electrolytes, a sharp anodic peak at 2.1 and 1.6 V is observed, which relates to the Al intercalation and deintercalation process. With cycling, it is evident that the peak current decreases significantly, and small shifts in both the cathodic and anodic peaks are observed. In comparison, the addition of IL leads to a broad wave peaking around 2.15 and 1.55 V which are related to Al intercalation and deintercalation in the Mn–O matrix. However, after 25 and 50 CV cycles, the decrease in peak intensity is much less compared to aqueous electrolytes. This indicates that the change in Al coordination in the electrolyte makes the Al deposition/stripping at the Zn anode and the intercalation/deintercalation at the MnO_*x*_ cathode easier. This is further supported by the galvanostatic charge/discharge analysis and microstructure of the MnO_*x*_ thin films. Figure 4b shows the first charge–discharge plot on electrodeposited MnO_*x*_ from which an increase in the capacity by about 35% is observed on the addition of EMIMTfO. The increase could be corroborated by the slower diffusion of Al ions over a potential range into the Mn–O matrix compared to a sharp intercalation potential observed in an aqueous electrolyte (see Figure 4a). Due to the slower diffusion of Al ions, the strain in the MnO_*x*_ thin film was considerably reduced, which was confirmed by microstructural analysis. Figure S9 compares the electrodeposited MnO_*x*_ thin film after one discharge–charge process in aqueous and aqueous–IL electrolytes. It is evident that in an aqueous solution, significant strain-induced cracking of the MnO_*x*_ thin film occurs as compared to that in an aqueous-IL electrolyte. After discharging and charging the cell (Figure S6a), the disintegration of MnO_*x*_ is observed, and after one discharge–charge–discharge process (Figure S6b), the MnO_*x*_ thin film disintegrated completely. In comparison, in the aqueous–IL electrolyte, after discharging and charging (Figure S6c), a lower number of cracks are observed, and after discharging–charging and discharging, the number of cracks increases, but complete disintegration does not occur (Figure S6d).

From the electrochemical and microstructural analyses, it is evident that the presence of IL in the electrolyte influences the Al intercalation/deintercalation process in MnO_*x*_ and improves the charge storage capability of Al in MnO_*x*_. However, due to the disintegration of the cathode during the charge–discharge process, significant initial capacity loss during galvanostatic cycling was observed. Figure 4c compares the cycling of Zn/Al-MnO_*x*_ battery wherein almost an exponential decay in capacity is observed in the first 10 cycles, which has also been shown by previous authors.^11,12,15^ However, in our case, as thin-film MnO_*x*_ was electrochemically deposited without any binders, the disintegration of MnO_*x*_ was substantial. After about 20 cycles, due to the disintegration of MnO_2_, insignificant capacity was observed when using 3 M AlTfO. However, a capacity of about 75 mA/g was observed when the aqueous + IL electrolyte was used. Balland et al.^15^ had shown that dissolution of MnO_2_ occurs in the triflate-based aqueous electrolyte and therefore to counter the dissolution, experiments were also performed with 0.1 M MnTfO added to the aqueous + IL electrolyte. Although a substantial increase in initial capacity was observed (Figure 4d), considerable decay in capacity took place, which indicated that disintegration of electrodeposited MnO_*x*_ might be the major cause. To understand the capacity fading and Al storage mechanism in MnO_2_, XPS was performed after the charge–discharge process.

**Figure 5:**
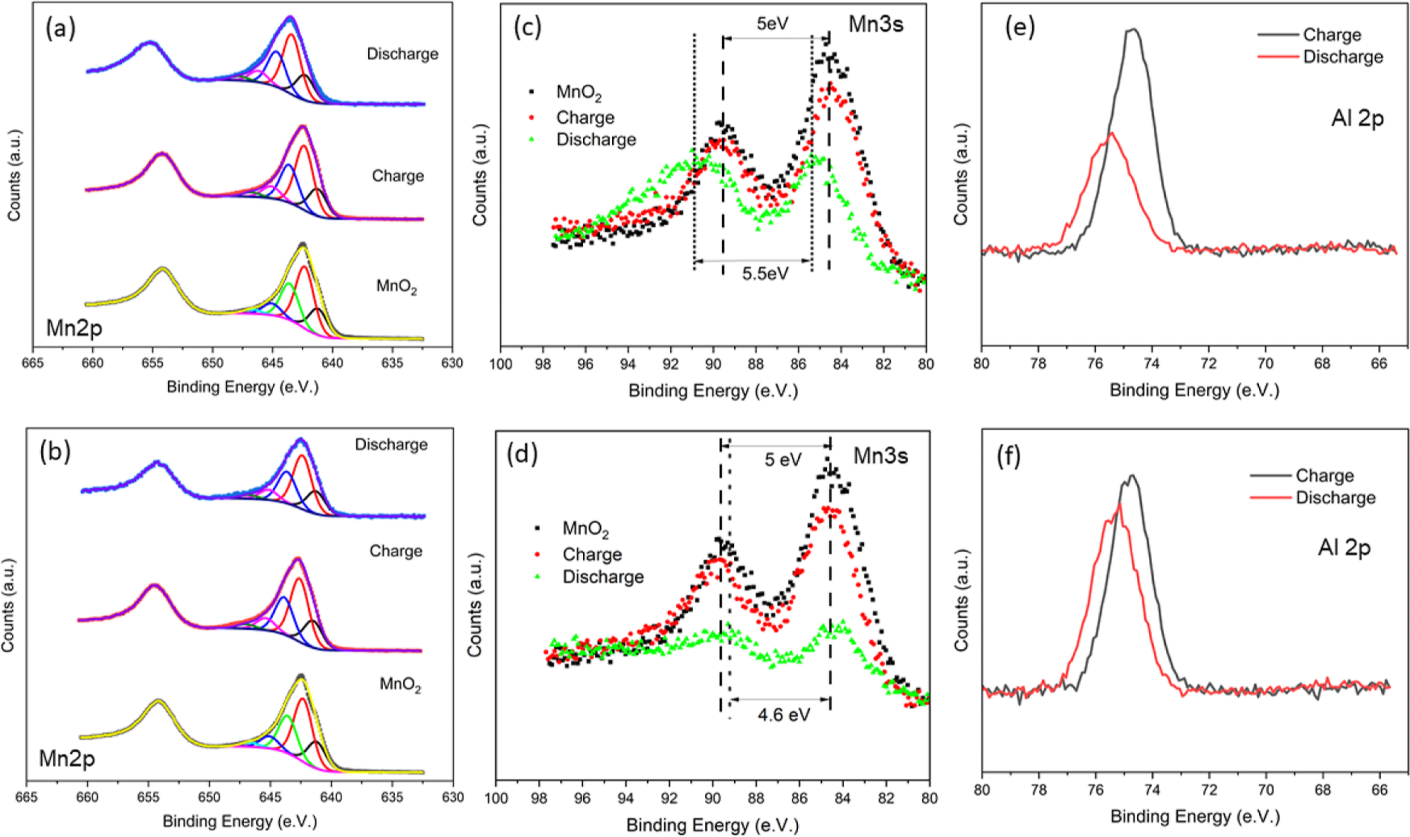
High-resolution XPS spectra of (a) Mn 2p for the aqueous electrolyte, (b) Mn 2p for the aqueous + IL electrolyte, (c) Mn 3s for the aqueous electrolyte, (d) Mn 3s for the aqueous + IL electrolyte, (e) Al 2p for the aqueous electrolyte, and the (f) Al 2p for aqueous + IL electrolyte.

Figure 5 compares the XPS spectra of MnO_*x*_ after charge and discharge processes in aqueous and aqueous containing EMIMTfO. The XPS survey spectra are shown in Figure S10 from which Mn, O, S, F, Al, and C were observed. The high-resolution Mn 2p_1/2_ spectra in Figure 5a can be discerned into four peaks at 642.3, 641.2, 643.6, and 645.2 eV corresponding to MnOOH/Mn_2_O_3_.^19,20^ Interestingly, after one charge and discharge process in 3 M AlTfO, no change in the Mn 2p peaks take place. A similar phenomenon is seen in Figure 5b, which corresponds to manganese oxide cycled in 3 M AlTfO + EMIMTfO. It has been shown previously that no change in Mn 2p takes place in Al-ion batteries.^12^ However, on comparing Mn 3s spectra in Figure 5c,d, it is evident that in the case of IL, there appears to be a shift in the Mn-oxidation states during the discharge process which indicates that some intercalation/deintercalation of Al^3+^ takes place. On comparing the Al 2p spectra (Figure 5e,f), it is evident that after charging, an Al peak at 74.7 eV is observed which corresponds to the presence of Al(OH)_3_. After the discharge, a shift in the peak to 75.2 eV is observed, which relates to AlO(OH). This clearly indicates that in the first discharge process, Al^3+^ along with H^+^/H_3_O^+^ enters the Mn–O matrix, and the charging takes place through proton intercalation. Reports have claimed that the storage mechanism of MnO_2_ in the aluminum battery is mainly due to Al^3+^ and H^+^ cointercalation which forms layered Al_*x*_MnO_2_·*n*H_2_O.^10^ Recently, Wang et al. used spectroelectrochemistry to show that the dominant mechanism for Al batteries is the intercalation/deintercalation of protons.^21^ However, in our case, we can see that during the first discharge, we have cointercalation of Al^3+^ and H^+^/H_3_O^+^, which leads to cracks in the electrodeposited manganese oxide.^22^ With a change in Al solvation in the presence of IL, the strain within the Mn–O matrix might have lowered, and the aluminum concentration was found to be higher which leads to higher capacity. The charge–discharge cycle then takes place via proton intercalation/deintercalation with the Al_*x*_MnO_2_·H_2_O complex and small amounts of Al^3+^. Finally, relatively stable capacity is achieved through the protonation/deprotonation process.

**Figure 6:**
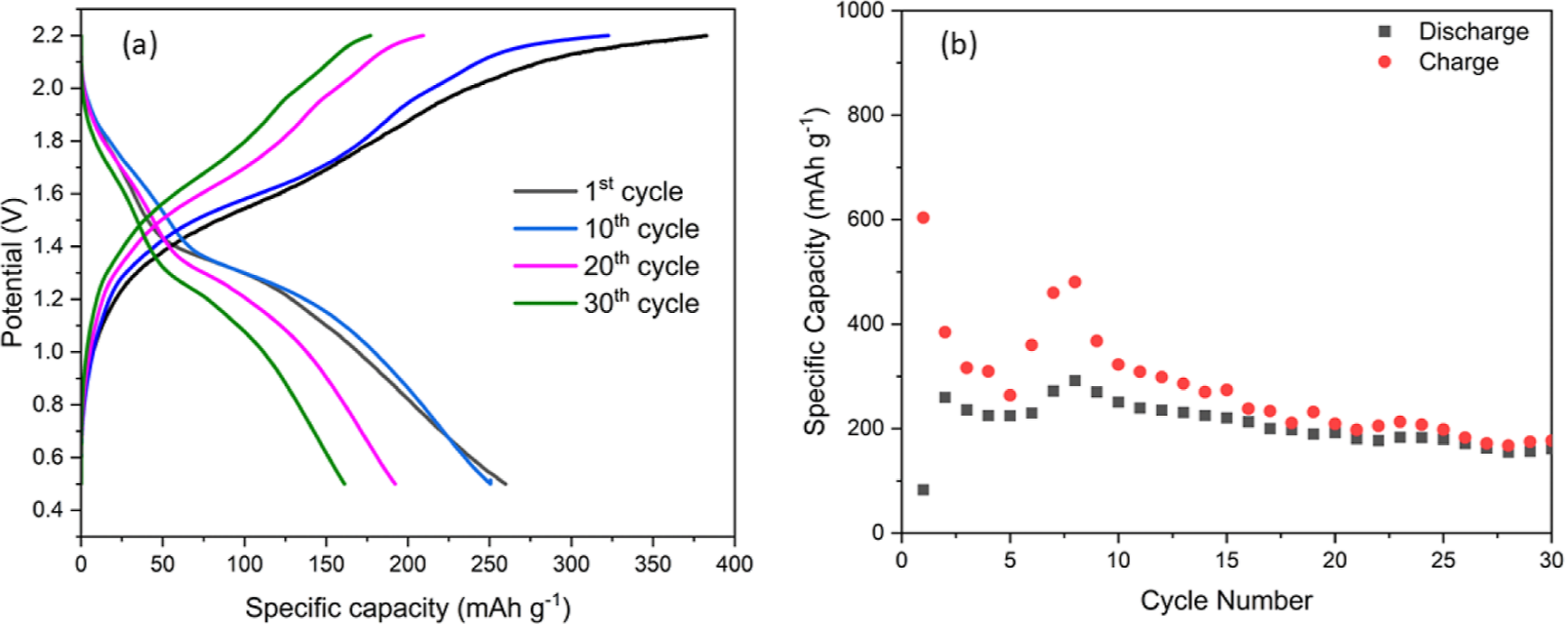
(a) Charge–discharge profile of MnO_2_-aniline composites in the aqueous + IL electrolyte. (b) Charge–discharge cycles in the ZnAl/MnO_2_-aniline battery in 3 M AlTfO + 50 wt % EMIMTfO cycled at 100 mA/g.

As strain-related cracks develop in the manganese oxide thin films, to improve the stability, polyaniline was coelectrodeposited with MnO_2_. It is known that conducting polymers not only provide a conducting backbone to the metal oxide but also accommodate internal strain during the intercalation/deintercalation process.^23−25^Figure 6a shows the charge–discharge profiles for Zn/MnO_2_-polyaniline that show a higher stability. An initial capacity of 250 mA h g^–1^ was achieved, which slowly declined with cycling. A consistent stability of >150 mA h g^–1^ was achieved for 30 cycles which clearly shows the promising nature of the aqueous + IL electrolyte with a modified cathode.

## Conclusions

For a successful aqueous-based Al battery, it is essential that Al passivation is relegated, and Al deposition/stripping can take place prior to the hydrogen evolution reaction. In this study, we have shown that ILs can significantly modify the Al solvation structure, which leads to improved Al deposition/stripping on Zn electrodes and resulted in a Zn–Al/MnO_*x*_ battery having a potential of >1.7 V. DFT calculations showed that Al coordination changes in the presence of IL which makes the Al intercalation into MnO_*x*_ over a range of potential, thereby inducing less strain within the Mn–O matrix leading to improved storage capacity. However, with cycling the strain within the matrix led to formation of cracks and lower capacity retention. The addition of polyaniline in MnO_*x*_ led to an improvement in both the storage capacity and capacity retention.

## Supporting Information Available

Additional CV cycles of Al electrochemistry on Zn and SEM and EDX analyses of the Al deposited on Zn; XPS survey spectra of MnO_*x*_; fully relaxed geometries and their coordinates and IR spectra, as obtained from the DFT calculations (PDF)
