# Supplementary material for: Modulating Aluminum Solvation with Ionic Liquids for Improved Aqueous-Based Aluminum-Ion Batteries
Source: ACS Appl Energy Mater. 2023 Nov 22;6(23):11874–81. doi: 10.1021/acsaem.3c01745 (PMC10716968; doi:10.1021/acsaem.3c01745)
Supplement: Supplementary file 1 — ae3c01745_si_001.pdf [file ae3c01745_si_001.pdf]

Supporting information

Modulating aluminium solvation with ionic liquids for improved aqueous based Aluminium-ion batteries

Abhishek Lahiri[1*], Shaoliang Guan[2,3] and Arunabhiram Chutia[4*]

[1]Department of Chemical Engineering, Brunel University London, Uxbridge, UB8 3PH, United Kingdom

[2]School of Chemistry, Cardiff University, Cardiff CF10 3AT, United Kingdom

[3]HarwellXPS, Research Complex at Harwell, Rutherford Appleton Laboratory, Didcot OX11 0FA, United Kingdom

[4]School of Chemistry, University of Lincoln, Brayford Pool, Lincoln, LN6 7UY, United Kingdom

Corresponding authors: abhishek.lahiri@brunel.ac.uk; achutia@lincoln.ac.uk

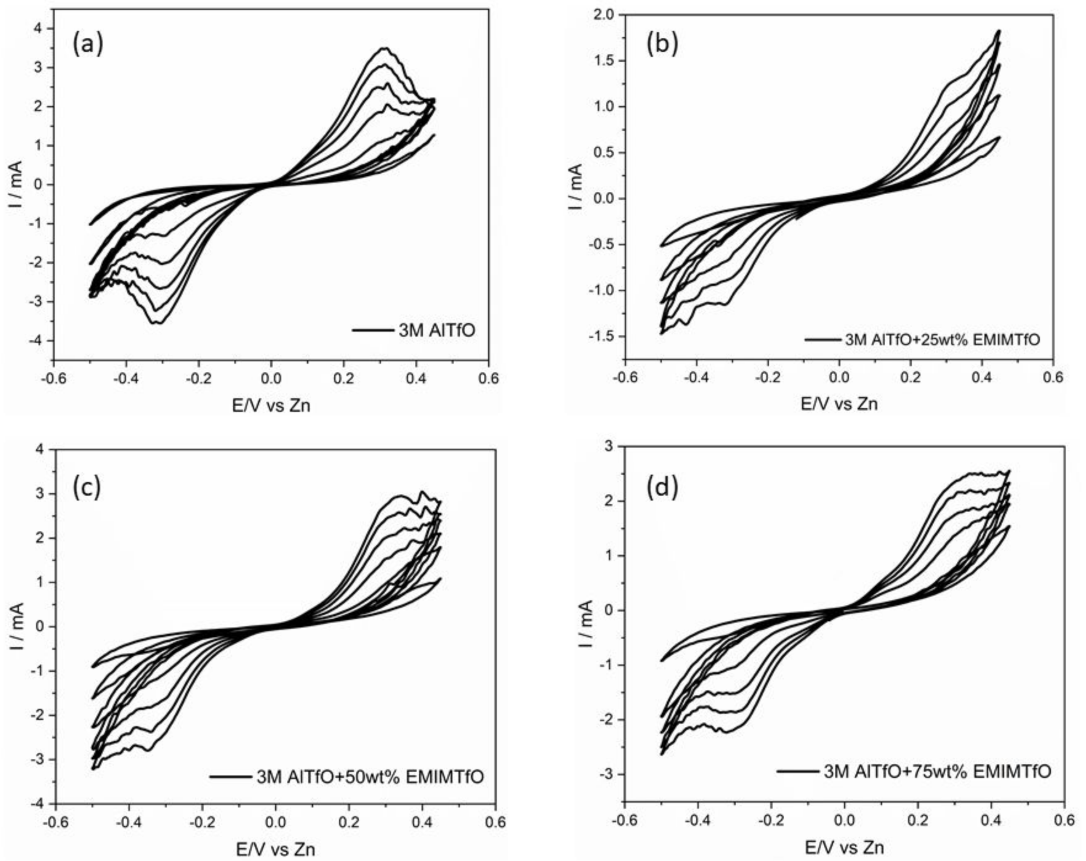

Figure S1: CV of (a) 3M Al(TfO)3 (b) 3M $Al(TfO)_3$+25wt% EMIMTfO (c) 3M $Al(TfO)_3$+50wt% EMIMTfO (d) 3M $Al(TfO)_3$+75wt% EMIMTfO on Zn

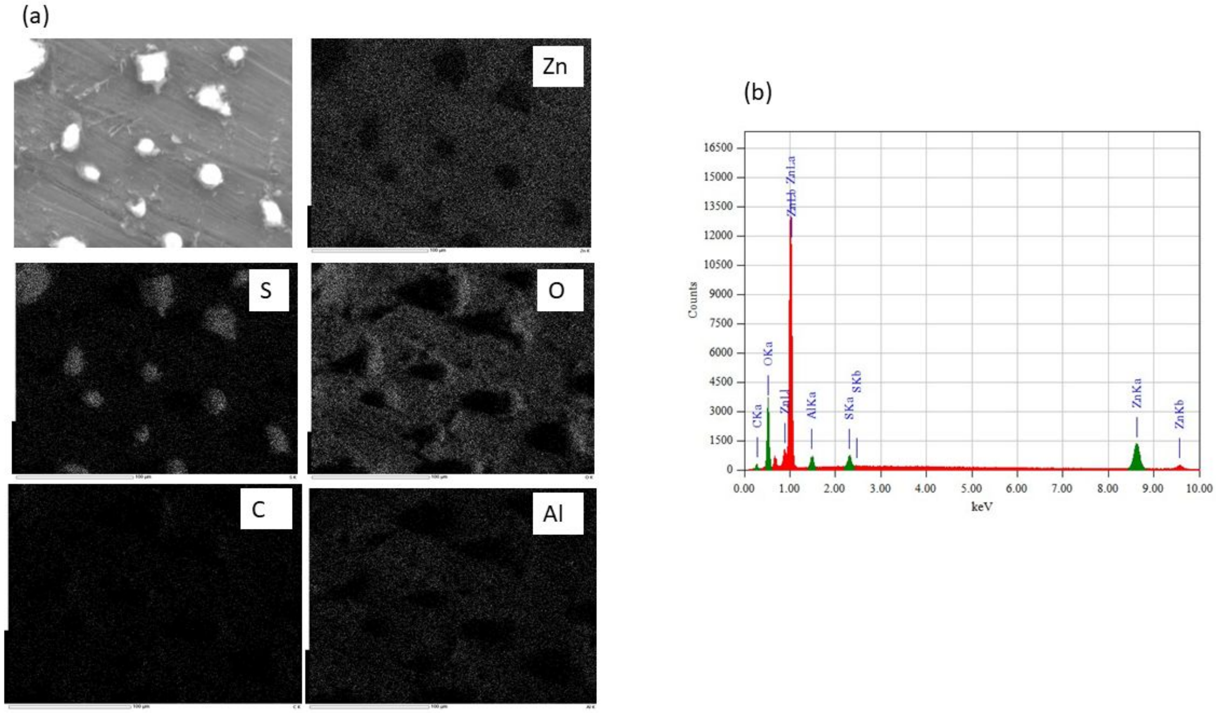

Figure S2 (a): SEM and mapping of the elements after Al deposition/stripping on Zn/Zn symmetric cell in 3M $Al(TfO)_3$ (b) EDX of Zn plate

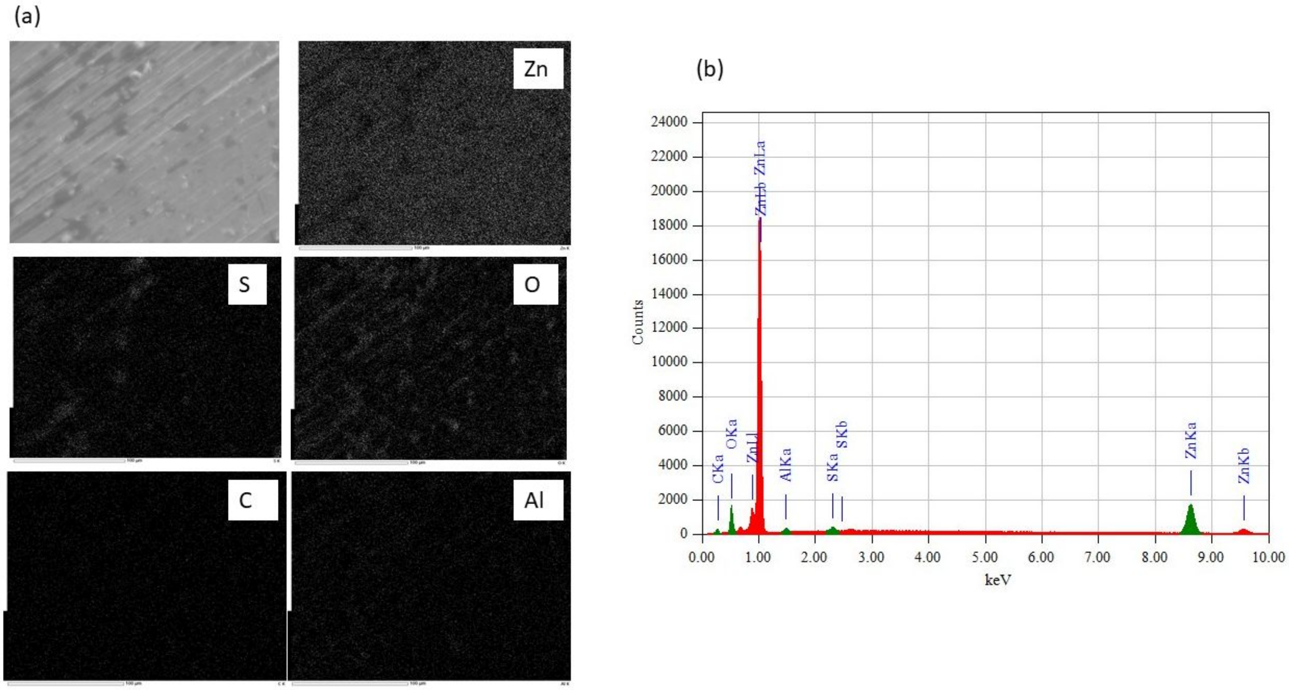

Figure S3 (a): SEM and mapping of the elements after Al deposition/stripping on Zn/Zn symmetric cell in 3M $Al(TfO)_3$+25wt% EMIMTfO (b) EDX of Zn plate

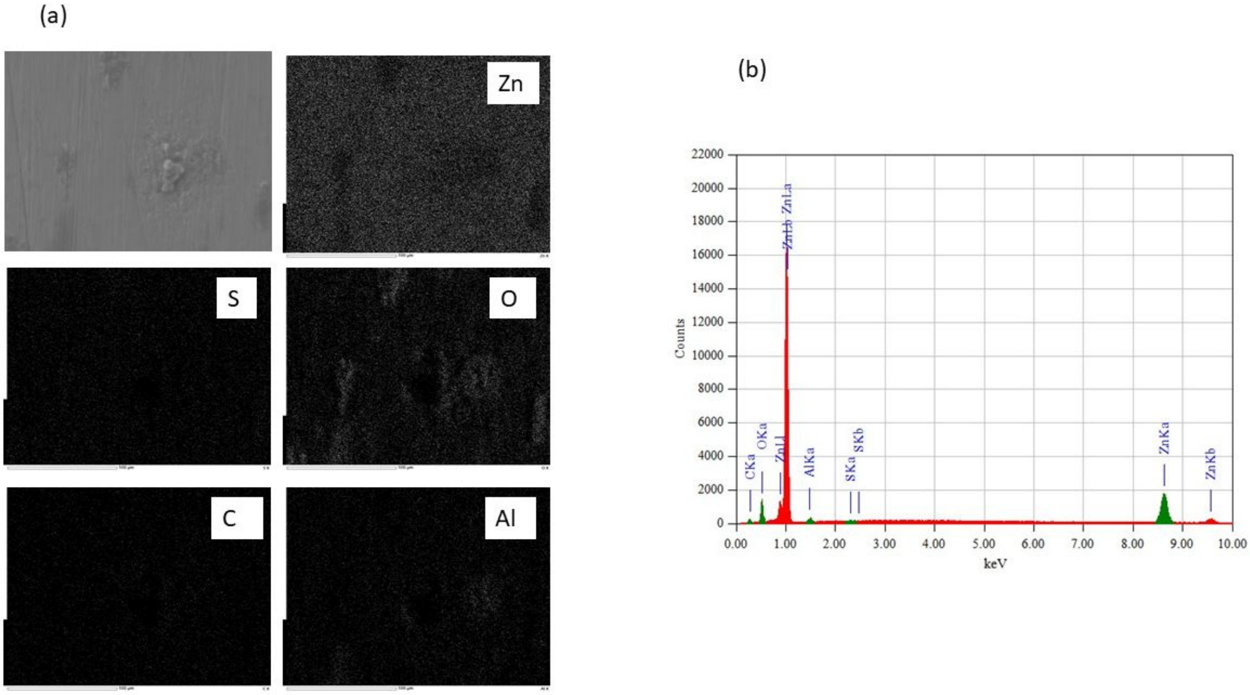

Figure S4 (a): SEM and mapping of the elements after Al deposition/stripping on Zn/Zn symmetric cell in 3M $Al(TfO)_3$+50wt% EMIMTfO (b) EDX of Zn plate

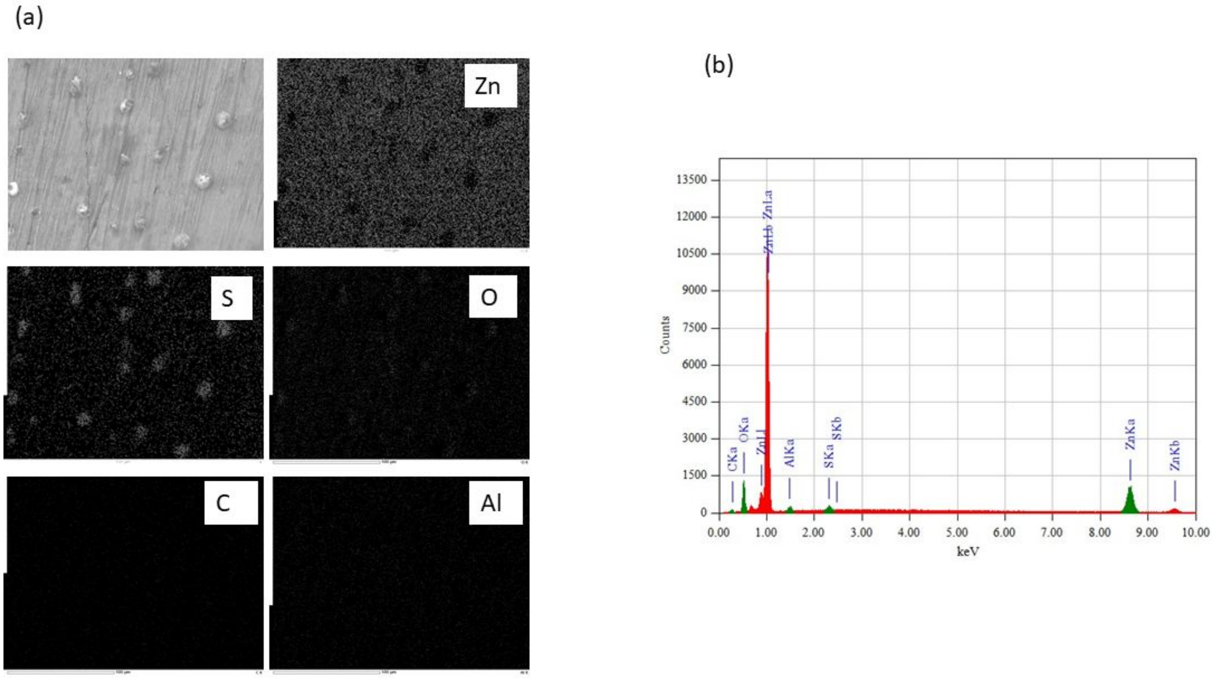

Figure S5 (a): SEM and mapping of the elements after Al deposition/stripping on Zn/Zn symmetric cell in 3M $Al(TfO)_3$+75wt% EMIMTfO (b) EDX of Zn plate

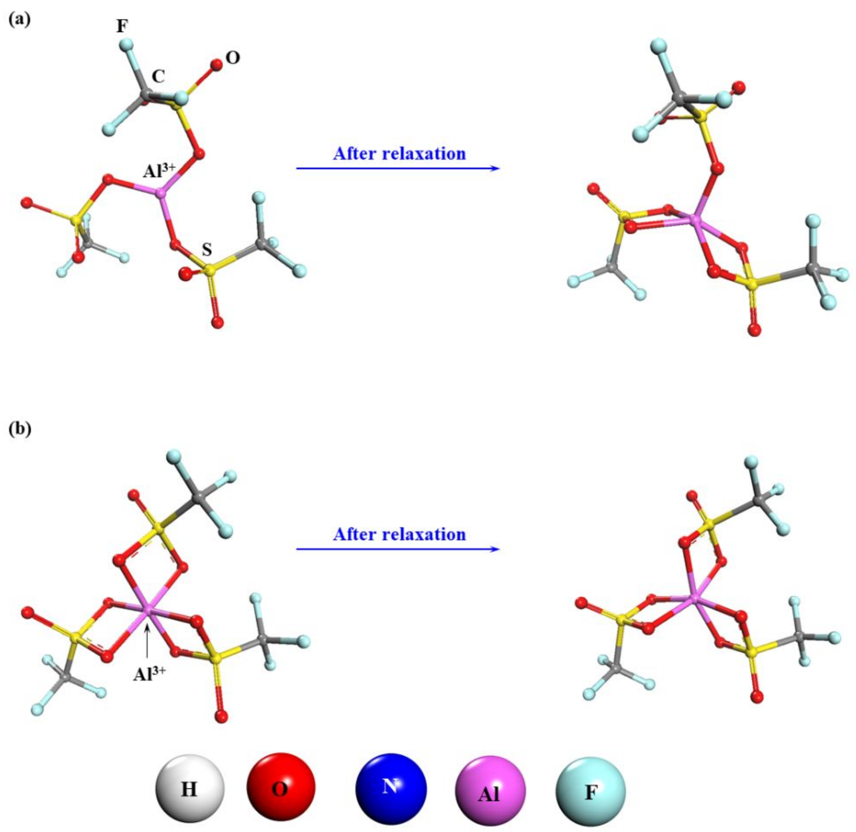

Figure S6. The geometries of $Al(OTf)_3.6H_2O$ (a) with all the TfO^- ions mono-coordinated to the Al^{3+} ion and (b) with all the TfO^- ions bi-coordinated to the Al^{3+} ion in the initial structures.

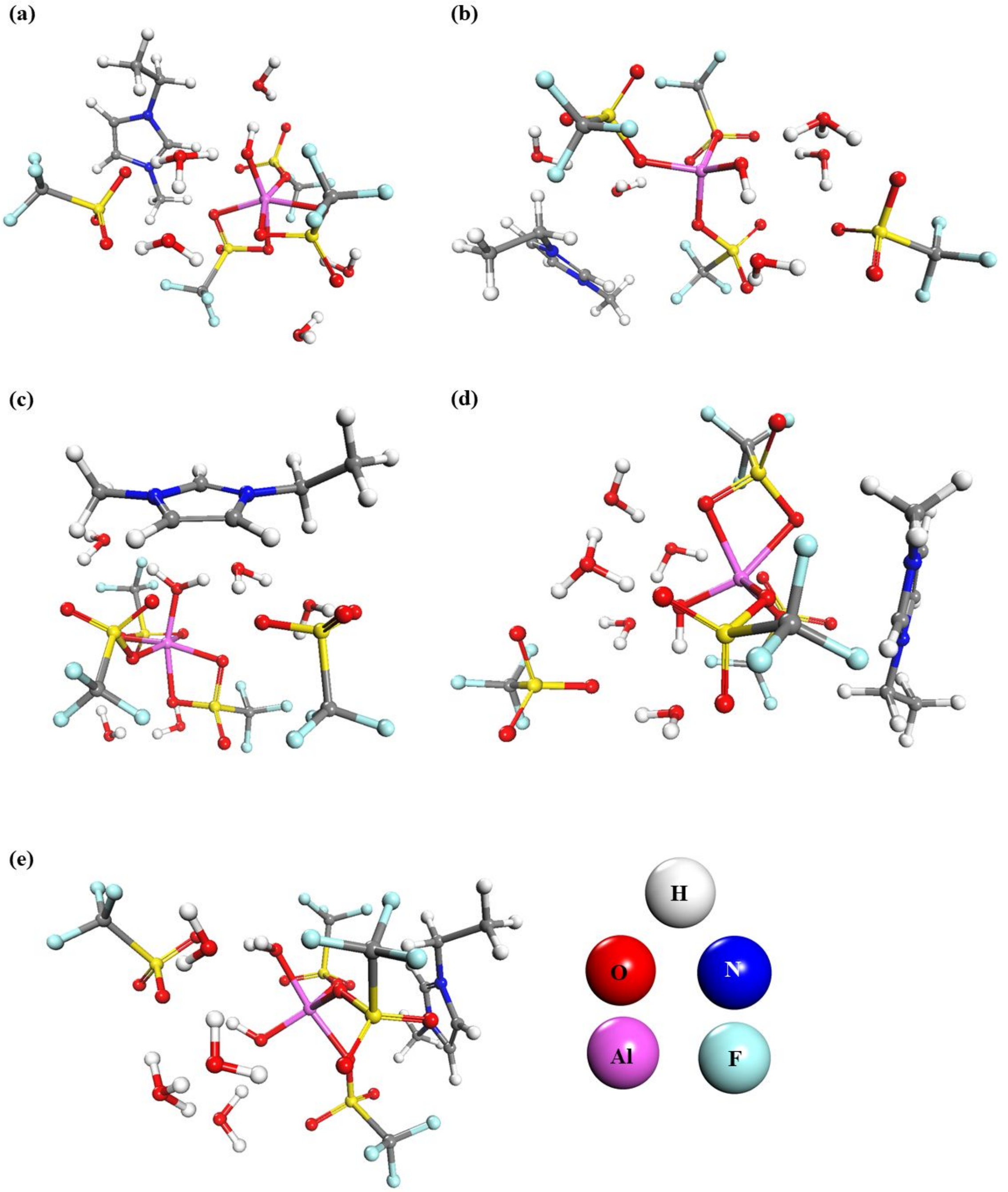

Figure S7. The fully relaxed geometries of $Al(OTf)_3.6H_2O$ systems with (a) $EMIM^+$ TfO^- ions close to each other (**M1**), (b) the $EMIM^+$ and the TfO^- placed away from each other (**M2**), (c) $EMIM^+$ TfO^- ions placed relatively further than M1 but closer than M2 (**M3**), (d) the $EMIM^+$ and the TfO^- ions placed away from from each other like M2 but with all the water molecules close to the TfO^- ion (M4) and (e) the $EMIM^+$ and the TfO^- ions are placed away from each other like M2 but with 5 water molecules close to the TfO^- ion and another close to the $EMIM^+$ ion (M5).

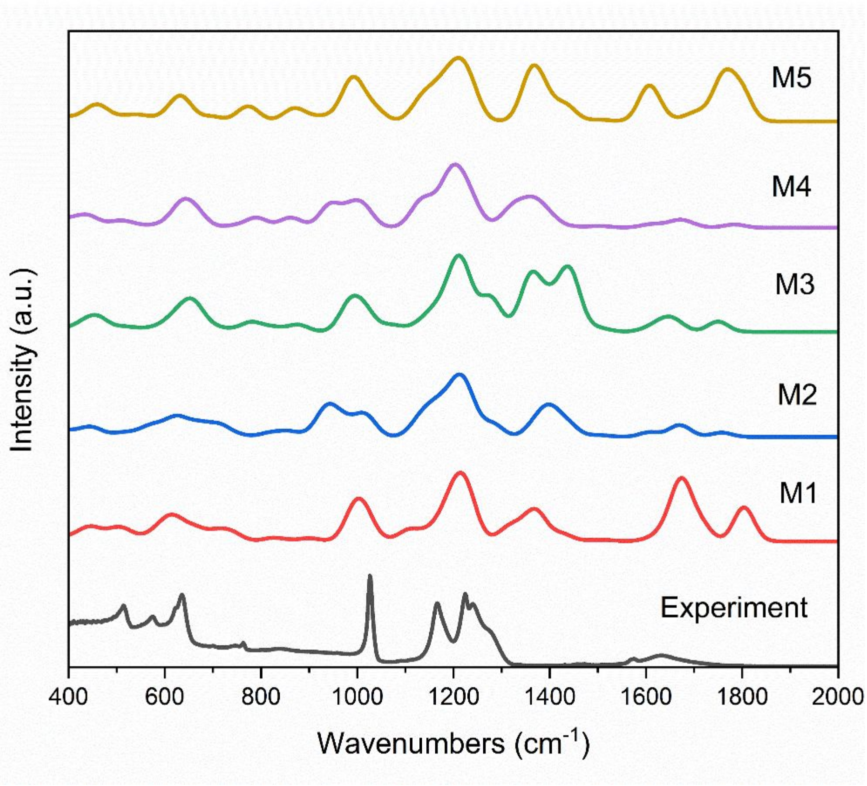

Figure S8: A comparison of the experimental IR spectrum and DFT-derived IR spectra for the M1-5 models.

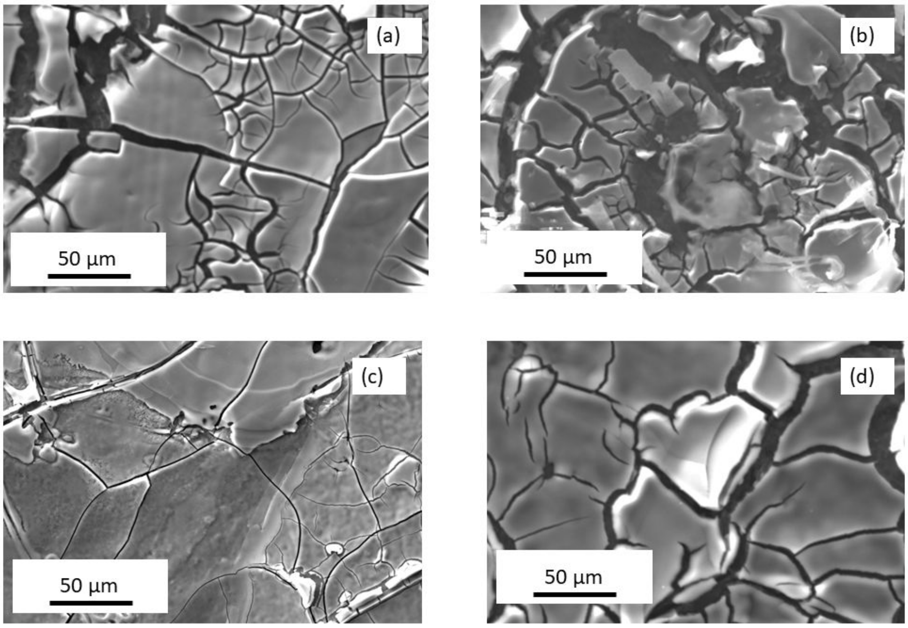

Figure S9 : SEM of electrodeposited MnO_2 after (a) one discharge-charge process in 3M $Al(TfO)_3$ (b) one discharge-charge-discharge process in 3M $Al(TfO)_3$ (c) one discharge-charge process in 3M $Al(TfO)_3$ +50wt% EMIMTfO (d) one discharge-charge-discharge process in 3M $Al(TfO)_3$+50wt% EMIMTfO

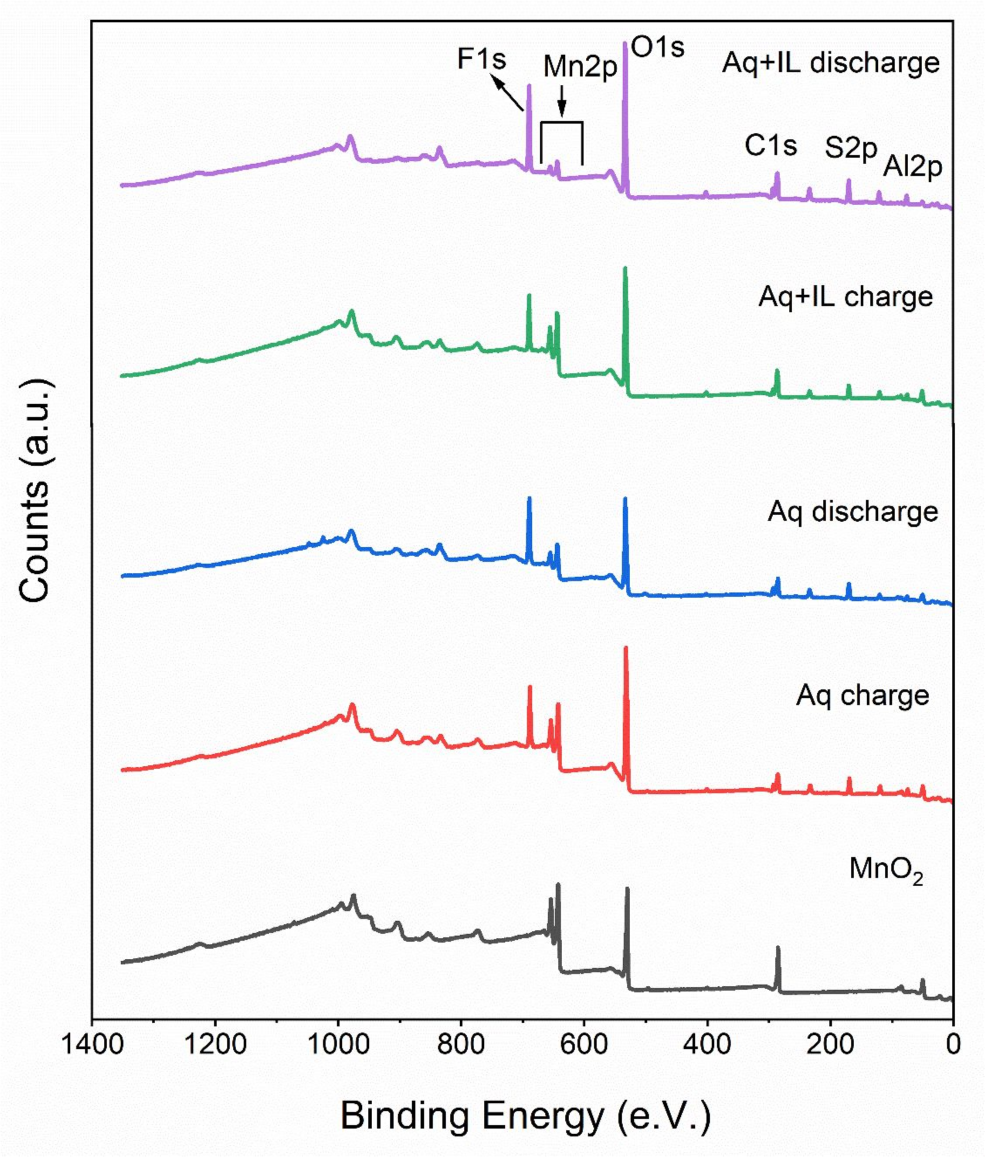

Figure S10: Comparison of survey spectra of MnO2 cathode in different electrolytes after complete charge and discharge states.

The fully optimised structures of all the models are included in *.xyz format.

Al(TfO)$_3$ 1st Model

```
25
Coordinates from ORCA-job bAlTfO3_B3_model-1
  C   -5.70146698194439     0.28754730168195     0.23800434755623
  F   -4.71748002975993    -0.58130340358394     0.47350121038668
  F   -5.92802406019119     0.36454867726539    -1.06455182914982
  F   -6.80040998583711    -0.09336611391986     0.87171011204480
  S   -5.18117395249934     1.97304728749869     0.87015472449906
  O   -6.23046648650565     2.87986345061607     0.60033697415282
  O   -3.82028777123264     2.18255601782007     0.25453553836338
  O   -4.75911570028921     1.72368891588557     2.29786986350790
  Al  -2.90731337460063     1.91185170148303     1.89891224794387
  C    0.32839942191028     0.63186883694651     3.11612474097808
  F    0.27238552063999     1.93406400056401     3.34667303023972
  F    1.35597183207868     0.34991156703710     2.32839773589223
  F    0.43450321183866    -0.03034453591106     4.26022799153704
  S   -1.25022198867079     0.07094649610961     2.26698202388467
  O   -1.14419579559460    -1.32113954094182     2.02757823515104
  O   -1.43950947078667     0.99201086673342     1.08899037434455
  O   -2.36661125382332     0.57277650626898     3.14731273031319
  C   -3.25090223185311     4.99848208352752     4.30888725165712
  F   -4.18146757553854     4.07672999093232     4.57961925662583
  F   -3.75332326821140     6.20855482151392     4.53361108355503
  F   -2.19962523672851     4.79856469444843     5.10264963366616
  S   -2.74602617937169     4.86803842943954     2.51759491731314
  O   -1.63843358044500     5.74285983251887     2.32776374648988
  O   -3.94817195258453     4.98451227240766     1.74719521592379
  O   -2.26021310999884     3.39893984365762     2.52330884312327
```

Al(TfO)$_3$ 2nd Model

25
Coordinates from ORCA-job aAlTfO3_B3_model2

C	-5.30065976689922	0.18858245732540	-0.40147536022640
F	-4.11471136214861	-0.39086518845772	-0.28029557623151
F	-5.46528362762302	0.62446052460323	-1.64511685898109
F	-6.25818840185981	-0.67794990423784	-0.09548648474502
S	-5.42468147163778	1.65326929144899	0.76315304668675
O	-6.73522422732815	2.17742046181134	0.62786051041428
O	-4.25472537492629	2.53477098386104	0.42390533111970
O	-4.98034452894513	1.14188389179673	2.10528513329742
Al	-3.36492107187232	2.21081285746661	2.10968428655391
C	0.00517326902325	0.89756433010008	3.11000941367568
F	0.16369239175380	2.18839540180646	2.84871313432388
F	0.79313327262081	0.17826697192768	2.32045658344393
F	0.29826576874979	0.65819617197907	4.38292144960091
S	-1.77315788537192	0.38683688998841	2.80651247784979
O	-1.84712300756745	-1.00705818345472	3.05621803227616
O	-2.11387369922256	0.89705273064520	1.43482143678502
O	-2.60426977238272	1.31698240490377	3.64408629472923
C	-2.07330870741880	4.93676194782084	4.32651686669761
F	-1.66442645149216	3.78634718850982	4.84759606040579
F	-2.86750565287194	5.56132936347494	5.18637556588688
F	-1.02288048231747	5.69725108250619	4.04130797184042
S	-3.02852947300991	4.62733410534417	2.74441546405951
O	-3.36838744815920	5.89852841142418	2.21675612074687
O	-4.12992651775939	3.67741356567511	3.11842874843750
O	-2.15928577133371	3.70928224173095	1.93052035135273

$Al(TfO)_3.6H_2O$

43

Coordinates from ORCA-job aAlTfO_6Wat

C	-5.71738838393600	0.39131353707784	-0.26326251496282
F	-5.21396035950801	-0.80399229213468	-0.16477315181785
F	-4.93642305197493	1.12186264224596	-0.99014894275260
F	-6.90248272859432	0.32615280903499	-0.84686423504562
S	-5.85223888525923	1.14148018478757	1.42607159246655
O	-6.32533742174098	2.46807334830777	1.23110565539099
O	-4.41477858963502	1.09832442481853	1.83154599197085
O	-6.63177658075252	0.25480182205697	2.19857019974945
Al	-3.23074581901567	2.01364739041873	2.90342884071240
C	0.34894394517323	0.66552832833764	3.19127754858809
F	0.42669851158981	1.91657905309264	3.62416622643509
F	1.22002005245057	0.44458857326385	2.22035381445790
F	0.57648833125111	-0.18097668177194	4.21411136717449
S	-1.38033897590703	0.27068011413959	2.54538647918248
O	-1.32328484607953	-1.04872449550619	2.02335712809021
O	-1.80363618008181	1.38695639098397	1.65471730502062
O	-2.29487547746088	0.50150784978202	3.73014234439877
C	-3.19619769635776	5.72215471768156	3.84801804403307
F	-3.90396557991111	5.14534353244916	4.81220996404939
F	-3.98570752355539	6.50124193598935	3.12509904309886
F	-2.21470706998285	6.44236896652818	4.39129418027781
S	-2.39690403605813	4.43794748367364	2.73972249673857
O	-1.47328789085072	5.15104888637235	1.92112763490061
O	-3.52576633472963	3.72152367554860	2.03501543147205
O	-1.91759275860982	3.37471819259691	3.65163107176070
O	0.45941739109241	3.08023649794500	0.64392076603530
H	0.41875807392636	3.94890155601118	1.05734438542266
H	-0.33204377646822	3.04606881344941	0.08993512472137
O	-5.65009147175034	0.12101089951684	4.88611434088918
H	-5.03079172259618	-0.62287872829027	5.06432998626803
H	-6.12778544463187	-0.11448864021301	4.07763170802792
O	-4.39663288506172	2.21667736413607	4.30217130988362
H	-4.90359052542651	1.36042155559341	4.59754566474379
H	-5.09186233525875	2.92462526495467	4.18428614808895
O	-2.19445072463339	3.00645239054700	-0.80321253528976
H	-2.80361515213928	3.56805253182059	-0.30057736434882
H	-2.44981609120698	2.13838222900054	-0.59553035510546
O	-3.66607521281096	-1.68685085517523	5.11651358146002
H	-3.31302532539527	-2.14650119768404	5.89684216762992
H	-2.94484592318373	-1.11888499828124	4.72568303066693
O	-6.27994101695351	3.94217851877975	3.64025923258561
H	-7.15173818395276	4.02389035403735	4.10073004496892
H	-6.47859432401209	3.62008605407669	2.71917924796136

Al(TfO)$_3$.6H$_2$O with EMIMTfO (model -1)

70

Coordinates from ORCA-job aM1

C	-6.34442281579552	2.63190843064650	-0.68209094479479
F	-5.49337555405586	1.61368665282885	-0.81947924868722
F	-5.78579182782474	3.73608308373244	-1.17108851401054
F	-7.46121670996063	2.36347017183992	-1.35386392170948
S	-6.74451856024300	2.86204670413276	1.12420134586916
O	-7.55612577544491	4.05087546309364	1.21537245463682
O	-5.37060150672410	3.10340364638762	1.67626205518456
O	-7.31041564036882	1.61864134432822	1.57758951717968
Al	-4.23514816790334	2.48489438907756	2.98504554079364
C	-2.98118815823635	-1.17553514582703	3.03951005250206
F	-2.14229638867624	-0.52820087210622	3.83940221221801
F	-2.31642148094095	-1.81205226788327	2.09470686041304
F	-3.69527136695540	-2.04051631217832	3.75669639634303
S	-4.20975472284584	0.00363929930186	2.24890988710440
O	-5.00515712823076	-0.80422546170057	1.38781594506538
O	-3.46552584560196	1.14980238081264	1.66006944595937
O	-4.89605757744832	0.68535356852984	3.40150817772614
C	-1.65942363223104	4.74144000810450	4.65011717008592
F	-2.84264189943004	5.06448274110089	5.15720246584827
F	-1.12861395799603	5.80040682503324	4.04642923542210
F	-0.85387162254464	4.32856722162521	5.62466436040810
S	-1.82065375373127	3.35730804195262	3.40222111676693
O	-0.50381524905757	3.14633197756156	2.90500402218916
O	-2.87313806125097	3.80473988552985	2.43518010970330
O	-2.51360918710087	2.24907770347710	4.11204401628980
O	-0.46657061038257	0.55188115024888	1.25155480976149
H	-0.00727020576883	1.13436378640455	1.86539495339732
H	-0.85586933509629	1.16066863482010	0.60367659979265
O	-4.74540692258178	2.19081728091751	6.55592734650353
H	-3.79465847614666	1.81826791858992	6.63818636344068
H	-5.32346842600978	1.36211772392955	6.56400551139470
O	-5.02980297736462	3.24015827403991	4.37090384388618
H	-4.87838526284910	2.69515629319699	5.59413503495807
H	-5.80315396129061	3.82143179288856	4.25106012107787
O	-1.93273249003171	2.61017760917377	-0.39897088148451
H	-2.02472364553126	3.33829306042556	0.22758493887630
H	-2.75092568898036	2.10838047504144	-0.29770571873113
O	-2.52431866887232	0.92567139967858	6.71121551916816
H	-2.92374950153368	0.03235633136659	6.78278428890676
H	-2.08933079619348	0.95928443865939	5.84984901426918
O	-7.38160735639823	4.69945157032972	3.98238655879548
H	-7.35806810901658	5.63299796227698	4.21902942885487
H	-7.57360422201313	4.66659320043756	3.02619410575562
N	-8.82947599722866	1.01818334806387	5.53488900969125
C	-9.31612302051088	-0.13271007963964	6.12064068493173
C	-8.93364614360454	-1.17005306135071	5.33541546041135
N	-8.22779303791959	-0.63908696125398	4.27974938506756

C	-8.16416839000469	0.68105474810632	4.43073139890879
C	-8.97719727528140	2.38474438759177	6.05592071799399
H	-9.86103166742158	-0.11957682263097	7.04585897283900
H	-9.06063254440929	-2.22951635541634	5.45506770267928
C	-8.36385504549893	2.54456723657712	7.44086210527442
H	-10.04413978885883	2.61531415881012	6.07184276252155
H	-8.50458199063731	3.05371786493861	5.33751121098939
H	-7.30002788372091	2.31739300278462	7.43179151690357
H	-8.84328551157577	1.89139528077115	8.17109022746273
H	-8.49812978109958	3.57499082108229	7.77256193593928
C	-7.57630329089584	-1.40505720170380	3.21780548146987
H	-8.28734153387073	-2.12438514334326	2.81595790857821
H	-6.72000382145436	-1.92530351232650	3.64033535118620
H	-7.26162665453547	-0.72536895973477	2.43378715283901
H	-7.66499645197637	1.35253617642418	3.75605034435103
C	-6.05708990635767	-1.65875338294917	8.50759883911660
F	-7.39702441379791	-1.65639287070410	8.63492414350780
F	-5.56127333639745	-0.71627907679953	9.32108253357985
F	-5.59939570487820	-2.84748743112117	8.90171724588637
S	-5.57826873820154	-1.30630728783399	6.74192849360397
O	-4.12572259429646	-1.30954131060597	6.76247069748671
O	-6.16282012019519	0.03337496704812	6.53100222721099
O	-6.21911210870654	-2.34786091661236	5.97719489243626

Al(TfO)$_3$.6H$_2$O with EMIMTfO (model -2)

70
Coordinates from ORCA-job cM2

C	-4.01793584662866	0.02421497786734	-0.43132751292983
F	-2.69436447655183	-0.09593786656953	-0.51826942824291
F	-4.39916835051808	1.03745325417794	-1.24210562126259
F	-4.59026189677306	-1.09218162769716	-0.86289235825812
S	-4.57313966531211	0.36893249709570	1.31342246681202
O	-5.94095046196719	0.76773029884716	1.18594237825978
O	-3.67931241545754	1.57440783961024	1.62566787228275
O	-4.18580869545610	-0.77394830279206	2.08052995730514
Al	-2.99863506630047	2.08878994230692	3.20266308241352
C	0.63703553535915	0.17793517188378	4.10503599102852
F	1.08076722667215	1.35826940940598	3.66817320741916
F	1.54275716117591	-0.75933726274475	3.83390669161014
F	0.43808506937060	0.23594906555892	5.41661582789860
S	-0.95351169055725	-0.24540150569760	3.22794278708030
O	-1.38647110308150	-1.51211735530308	3.73554926627188
O	-0.68619735914258	-0.10134386729641	1.82116376706242
O	-1.82568802110954	0.88178182002225	3.75361459876394
C	-2.22098860419231	5.91855323134462	3.61687435260139
F	-3.24585430081452	5.55848523499879	4.37741295139045
F	-2.68666297143010	6.36771505735629	2.43948651985481
F	-1.54096677061564	6.89384654982203	4.21125358462011
S	-1.08204213041432	4.46712665384480	3.32557138809218
O	-0.11038356538665	4.92812962310700	2.37685268348794
O	-2.05643315927024	3.50557265649390	2.62030444415155
O	-0.69017614180500	3.97026936132474	4.60553111340126
O	1.35349784883716	4.29896776613982	-0.18887581758744
H	1.50634652820332	4.66483989625578	0.68986891189396
H	0.85847154760635	3.47771071193846	-0.01990707915502
O	-4.34067284938116	0.64307358975245	6.35254047447328
H	-4.07406043264441	-0.24163036828167	5.86976828703188
H	-5.34666929535940	0.53096746597277	6.51844278771740
O	-4.15763756922047	2.34258831624128	4.42094268809411
H	-4.24867902846500	1.37864051680950	5.64964685325909
H	-5.07168931760589	2.58704990837318	4.11238615729388
O	-0.64535281700111	2.25877035798522	0.18535114969828
H	-1.15377822581363	2.71798838796397	0.86634055979529
H	-0.53728188318456	1.36493580393456	0.54999990317387
O	-4.00167997459080	-1.48363687073051	5.05510770176831
H	-4.77797079621816	-1.37176243558157	4.47310720756564
H	-3.22675638810519	-1.64487141496074	4.49564057621645
O	-6.55545094589536	2.82523619075646	3.30397198958678
H	-7.27907427568612	2.47189528822587	3.85623617342687
H	-6.55648230446165	2.22301318625880	2.54429303407616
N	-1.60682192758453	4.37904887720368	-1.64404393950138

C	-0.49613143456196	4.33478834799057	-2.45640923110741
C	-0.67307519808896	3.29618235572688	-3.31153338101924
N	-1.89113323796495	2.72178005463028	-3.00809804019341
C	-2.42833501247598	3.39138916811022	-1.98770133831684
C	-1.80988234682141	5.31281535208414	-0.52333060514411
H	0.33883867147121	4.99325692707162	-2.30693597504346
H	-0.04195430247145	2.91136146398291	-4.09175725004937
C	-2.08865814203350	6.73002246402819	-0.99908405974384
H	-0.90846018954492	5.26631564321530	0.08398904158032
H	-2.63657217795084	4.92298966376121	0.06884267719192
H	-2.99447812000643	6.77839916860853	-1.60569968249891
H	-1.25443724397441	7.11955203909233	-1.58424798931621
H	-2.22048419384594	7.37499091570796	-0.13029650375602
C	-2.43937308257484	1.52113686642076	-3.63440193073239
H	-2.00761894431094	0.63532932969918	-3.17076060058589
H	-2.20169830027235	1.53856993355287	-4.69565025900117
H	-3.51725966311181	1.50585057926063	-3.50555661018718
H	-3.35753562296686	3.14993090023878	-1.50443296988529
C	-8.49513178498576	-1.45242952997640	5.33991149373316
F	-9.46898951585356	-1.22094829820967	6.22386161246053
F	-7.76454520361459	-2.48917128590413	5.76896267520905
F	-9.04944006139929	-1.78088052181608	4.16750431497452
S	-7.41050159666726	0.04517364385559	5.12394987100381
O	-6.40171865981057	-0.38420088066728	4.16459855284631
O	-6.86539852765512	0.27507373216913	6.47323509302624
O	-8.31088630172359	1.08115193613691	4.65881346460907

Al(TfO)$_3$.6H_2O with EMIMTfO (model -3)

70

Coordinates from ORCA-job bM3

C	-6.09399479754016	0.38895548999239	0.49435298782490
F	-5.32981767320328	-0.65045248446012	0.79550268210343
F	-5.87187919189124	0.76187465359524	-0.76301077291900
F	-7.38294033153825	0.04732002580202	0.62789897315601
S	-5.75273086029874	1.79296532875182	1.66852936552280
O	-6.56265502043202	2.90463225845807	1.21440797964154
O	-4.29197012388251	2.02323192895567	1.40860124964572
O	-6.02226456191775	1.29171129158952	2.98669246617392
Al	-2.90149365127747	2.75666744069072	2.37863095499573
C	0.38199501871257	0.76690914252087	2.64076191855959
F	0.70732488510749	1.96977641199586	3.07374191180012
F	1.20224154508473	0.36046306820432	1.69500766293811
F	0.42155322015101	-0.09537027431579	3.66867328104070
S	-1.38930473879932	0.72898371148187	2.01502248689610
O	-1.60266994717152	-0.57318577678970	1.49434754205154
O	-1.61818085548575	1.90246381771630	1.11504047888532
O	-2.19517357312771	1.16646175529495	3.21494505362862
C	-2.26867394711439	6.51661327168218	2.88046426310820
F	-3.21741932745464	6.17930519366477	3.75727810093478
F	-2.77595347301803	7.38886000492740	2.01023019305867
F	-1.24854943012300	7.06830922920162	3.52431641494632
S	-1.67634040244854	5.00580113298662	1.95097667353252
O	-0.71540689949402	5.48810622212127	1.01379371960193
O	-2.93464901703288	4.41107877956133	1.37883465497325
O	-1.28499879666545	4.00833080115727	2.96209173655375
O	1.30948700263577	3.09215199812336	0.35710313122110
H	1.25003433694979	3.95203749914012	0.78618649601196
H	0.70260428806033	3.16647533425141	-0.39794666558273
O	-4.35116619342506	1.46849609116749	5.42704309846452
H	-3.49453244813330	1.14635951018592	5.83615141581000
H	-4.67768302573227	0.66287334911545	4.95529829200600
O	-3.83802273024165	3.23891910861272	3.84626645326161
H	-4.09295886301375	2.42738007687122	4.57434993456173
H	-4.60415468830078	3.84724679067637	3.73785111646884
O	-0.97431329379933	3.37791758997093	-1.45380465355682
H	-1.26737909386018	4.22379578605303	-1.09453402998659
H	-1.44717559614666	2.71156915701250	-0.93897188462834
O	-2.15727313840443	0.28622665840074	6.30395440555805
H	-2.57064460285230	-0.59854698049818	6.41074160177558
H	-1.58506346382243	0.21408110523553	5.53069881408301
O	-6.05888470068838	4.75127920019968	3.30641772147729
H	-6.35568431085553	4.33454951171191	2.47496539702775
H	-5.88652401578944	5.67749081149502	3.10343509773920
N	-7.27071418461339	0.25188920695929	6.37250441748535
C	-7.85960619696545	-0.64908239282182	5.51097827544290
C	-8.46552884270485	0.07542527529895	4.53601151440815
N	-8.23075206796433	1.40559249750912	4.80880279300762
C	-7.49614533394018	1.48337264718131	5.91627410069575

C	-6.43958305255576	-0.06431316529203	7.55198697652052
H	-7.72994644369485	-1.71034514278221	5.62143079916534
H	-8.99622339813260	-0.23868442493117	3.65623993233524
C	-6.98076364364744	-1.23927633631193	8.34755739194376
H	-6.40718368306039	0.84373977794648	8.15357366423432
H	-5.43090917941625	-0.26927496138364	7.20058135609032
H	-6.91887674277466	-2.16175536000336	7.77159516683931
H	-8.01108563787424	-1.06897391281716	8.66506572524900
H	-6.36132827235908	-1.36906432135450	9.23530985835483
C	-8.66843208096566	2.53461316462013	3.99493462990945
H	-9.72491786374183	2.73328561109504	4.17311919683334
H	-8.50149677708087	2.29833059983391	2.94762804196312
H	-8.07212678563063	3.40887397081845	4.24522896253514
H	-7.11799486108911	2.38929991728000	6.35340647458471
C	-3.66862076174260	-3.23034861422017	4.13522395789308
F	-2.56203865534331	-2.55506034428525	3.78749882572793
F	-4.37226419378909	-3.49341114720460	3.02917294179853
F	-3.29974527818248	-4.38948072420590	4.68919132211593
S	-4.67580726633442	-2.21373975850242	5.32916141512622
O	-5.81670078633421	-3.03959463521067	5.66721250181038
O	-5.00357889250308	-1.01645973184982	4.55166593470686
O	-3.75457262927360	-1.95664271787938	6.43549610285196

$Al(TfO)_3.6H_2O$ with EMIMTfO (model-4)

70
Coordinates from ORCA-job aM4

C	-4.72562948438862	0.09195930584521	-1.49844810321485
F	-3.71289362414176	-0.76093287198675	-1.68490655229280
F	-4.41560934229610	1.24124649062954	-2.14292125738780
F	-5.82503914192753	-0.40778864993410	-2.03989109201261
S	-5.00087529596820	0.40470931143669	0.32039832371381
O	-5.95872048056380	1.46938354829730	0.39422803992460
O	-3.59143490000809	0.90714322239993	0.66606467722747
O	-5.27544635735167	-0.87367377349698	0.89130817781883
Al	-2.91917969463811	1.10065726749831	2.33825979400594
C	0.31342881125740	-0.65140404087588	3.53420076815510
F	0.07350466423502	0.28694809617430	4.43500165614187
F	1.54309594254345	-0.50799410333473	3.04966136063491
F	0.19083870801311	-1.85460771872281	4.09751190162483
S	-0.91450755559159	-0.53764444096880	2.12778940699459
O	-0.47084993373438	-1.45553079252123	1.13261572741611
O	-0.98375457807786	0.90800098525285	1.75256742175165
O	-2.24690014025743	-0.73353413350348	2.75911026613491
C	-3.28588082527783	4.59809260426342	4.31804475601282
F	-4.02574040467286	3.71341568978961	4.96330849111088
F	-4.03712244388273	5.25533664268116	3.42839045224807
F	-2.77587012232880	5.47284579538204	5.18499417872607
S	-1.87450220308274	3.75418539824858	3.43153373608814
O	-1.15464388921850	4.81363018687043	2.78058675430236
O	-2.60987232754261	2.90868673651956	2.40783577218093
O	-1.20068065336663	2.93538280359821	4.40327273903397
O	-2.04697938366088	0.96037698489385	6.48742724784220
H	-1.67363049861055	1.57737760457832	5.84507881194096
H	-2.90266554869515	1.35674424684990	6.77654346842440
O	-4.84801574478757	-1.15342030456737	4.67901961175079
H	-4.03066287511120	-1.37954469787277	5.25059931017585
H	-5.68654664506061	-1.02360109696983	5.23261924468384
O	-4.18549924783739	0.93804808552398	3.49186431468319
H	-4.62114738410118	-0.27477265211931	4.15670246219243
H	-4.89443295690445	1.63121408597202	3.47856548946752
O	-4.44165311686852	1.95216761196456	7.28131209962553
H	-5.21617395939091	1.87698652409635	6.69074945288442
H	-4.76825890497543	1.68673604243989	8.14703880578177
O	-2.71253365992872	-1.54365405672936	6.01464206362701
H	-2.35161809921006	-0.62647029991916	6.18228619384177
H	-2.01861627269242	-2.06305563575031	5.59771152107580
O	-6.20284834383761	2.67702413164986	3.27045828027505
H	-6.68034708301487	2.45197580548401	4.09258634275866
H	-6.73380010813254	2.35466980608869	2.53173897091149
N	-1.74171311036706	4.16865740633261	-0.31548171869934
C	-0.40764250209302	4.11159182602328	0.02802447683642
C	0.06847445349465	2.92360941680416	-0.42404605885653
N	-0.97998398932130	2.27078199728478	-1.03594629916643

C	-2.06283580890730	3.04202978481472	-0.94960310232675
C	-2.71904758958207	5.22039124993145	0.04001367728399
H	0.07266810937558	4.88562918003750	0.59271679110852
H	1.04459317578644	2.48171016474756	-0.34498684575826
C	-2.09152058334959	6.59924077490234	0.12433710651109
H	-3.16472967423436	4.93539148022891	0.98994407333758
H	-3.49413731495788	5.18671260458823	-0.72545824601493
H	-1.58373084395330	6.86815119526377	-0.80346858009865
H	-1.39204202311196	6.66098639888569	0.95662341005112
H	-2.88097810082208	7.32875734003115	0.30557470253283
C	-0.92647066674780	0.93214815919545	-1.62008035584113
H	-0.86016719356451	0.18906269061409	-0.82844160377222
H	-0.05655278851373	0.86201523194115	-2.27004139121417
H	-1.82755374260601	0.76232503465325	-2.19973266767975
H	-3.04165832217304	2.78514726038789	-1.30830230517128
C	-8.02764315126226	0.71990365191178	7.88847466599200
F	-8.62010166137530	1.89059919251362	8.13919613034055
F	-6.86499082513936	0.68817630325841	8.57684753459720
F	-8.80503488471302	-0.26147479936278	8.34569129313106
S	-7.70575416567928	0.52224524048600	6.06171197192484
O	-7.02588176520130	-0.77620310119999	6.00162867781788
O	-6.80002774282117	1.64574755557231	5.77041070955961
O	-8.99490218306889	0.59526101499597	5.43571286529047

$Al(TfO)_3.6H_2O$ with EMIMTfO (model-5)

70
Coordinates from ORCA-job dM5

C	-6.18978036741948	3.19631577017087	0.19487684415641
F	-5.32254041646652	4.10599580148166	-0.30815787851984
F	-6.57748798364069	3.61035005043849	1.39568141230852
F	-7.24391583672885	3.13753003246943	-0.61311324759721
S	-5.36724563171339	1.52086070642707	0.26411082691682
O	-6.27210717798767	0.64952090892132	0.94413136220684
O	-4.13590561196124	1.84203893109946	1.09039802550284
O	-4.99605935586670	1.27116452599808	-1.10236972676084
Al	-3.54257045775905	1.35934942739616	2.75192004804398
C	0.34737255219452	-0.57835822354679	1.92926726325520
F	0.90987025943318	0.45395155603500	1.28000954716481
F	0.98012886528756	-1.69670289023161	1.56375279939881
F	0.50936941980262	-0.39564052368704	3.23707487843448
S	-1.46142192560473	-0.75095291689356	1.50515091573904
O	-1.88844715247919	-1.89064953341463	2.26788114660172
O	-1.51041127621463	-0.82823068850015	0.06946042797443
O	-1.99430201624449	0.55465532759868	2.01748994698379
C	-1.30810141193051	4.45208805486193	4.92931325588440
F	-2.07822193541341	4.18967285711129	5.98849232366038
F	-1.90035974530839	5.39734301797239	4.18992784804906
F	-0.13074536547567	4.90747727215423	5.35110907995407
S	-1.04636524014687	2.90678360493635	3.91881082249959
O	-0.17838026768235	3.29982533854203	2.84645026700315
O	-2.46470801434672	2.67950433589167	3.46126082282433
O	-0.59921315651378	1.90506409170811	4.84960312528317
O	-2.38196115461859	0.86503256494989	6.82199220348318
H	-1.66762082120920	1.02503773664924	6.18148021958371
H	-2.98573883094534	1.62669986577573	6.72513720814133
O	-3.44287584397326	-2.39438710472208	4.56634906632610
H	-3.58209634442050	-1.82269879097603	5.78043565070979
H	-2.61415622070772	-2.78483858773291	4.26409938243712
O	-4.05978142190712	-0.07444933073231	3.58020890589127
H	-3.60504898712278	-1.61343282507077	3.96141433736485
H	-4.86222778051980	0.00525847697914	4.10722657811978
O	-4.51542006315790	2.58250318868569	6.25185242764312
H	-4.89741941107517	3.25331418917848	6.82872404131792
H	-5.12016665041777	1.80448057569508	6.28716154808450
O	-3.83252734304574	-1.23943583414411	6.67621138022550
H	-4.73698125703359	-0.86598410526585	6.53483151345047
H	-3.19994693131723	-0.43059715229022	6.73996418534944
O	-4.95137139128162	2.47123964527406	3.55240025504914
H	-5.87411368613978	2.12300492220124	3.63522203651138
H	-4.71957066320173	2.75373993851515	4.46596964767287
N	-1.68172203489197	3.43547758752257	-0.35293049365101
C	-0.49642586570485	2.74049195541376	-0.21115568290050
C	-0.54922412786806	1.69296796150194	-1.07013846547148

N	-1.76455565747935	1.75441359481931	-1.71631033760381
C	-2.42950217834044	2.81610772593910	-1.26612512907479
C	-2.12148482217647	4.58473895040090	0.45603769607886
H	0.23910716671128	3.01097188293902	0.52092345841160
H	0.13702532416521	0.88454981079378	-1.23204964975708
C	-1.05838181006498	5.66479945355420	0.56013560507705
H	-2.38786911561914	4.20526864764041	1.44089864296915
H	-3.02813579867758	4.96328611628592	-0.01116051742192
H	-0.76520557098826	6.03006182982187	-0.42538737280892
H	-0.17761860452103	5.29988445022545	1.08651775390604
H	-1.46095540447825	6.50076265169410	1.13303401951837
C	-2.27602094313903	0.75334827341416	-2.65106738338615
H	-2.26340080435988	-0.21331008991947	-2.15255442195827
H	-1.64874617898882	0.73238894560324	-3.54108865833569
H	-3.29883358614913	1.00600908575261	-2.91136476512567
H	-3.42534000808298	3.09207583519084	-1.55605183477753
C	-8.10016247884584	1.38391207210997	6.88957916691741
F	-7.37685216114041	2.48310426993141	7.20765380988227
F	-8.12534343114863	0.58572697444483	7.95794496692395
F	-9.33928162384811	1.76995383337511	6.60259930484322
S	-7.31827074845650	0.50959338851037	5.43931509568410
O	-8.07543653550162	-0.68705136023746	5.24630851810674
O	-5.92804466282600	0.29635050385906	5.94382617177353
O	-7.33085428528938	1.51367144149861	4.38418777784442
